# Influence of ergosterol and phytosterols on wine alcoholic fermentation with *Saccharomyces cerevisiae* strains

**DOI:** 10.3389/fmicb.2022.966245

**Published:** 2022-09-08

**Authors:** Giovana Girardi-Piva, Erick Casalta, Jean-Luc Legras, Thibault Nidelet, Martine Pradal, Faïza Macna, David Ferreira, Anne Ortiz-Julien, Catherine Tesnière, Virginie Galeote, Jean-Roch Mouret

**Affiliations:** ^1^SPO, Univ Montpellier, INRAE, Institut Agro Montpellier, Montpellier, France; ^2^Lallemand SAS, Blagnac, France

**Keywords:** wine yeast, sterol limitation, high sugar content, yeast membrane, oenological fermentation, sterol type

## Abstract

Sterols are a fraction of the eukaryotic lipidome that is essential for the maintenance of cell membrane integrity and its good functionality. During alcoholic fermentation, they enhance yeast growth, metabolism and viability, as well as resistance to high sugar content and ethanol stress. Grape musts clarified in excess lead to the loss of solid particles rich in sterols, resulting in sluggish and stuck fermentations. Two sterol sources can help *Saccharomyces cerevisiae* yeasts to adapt to fermentation stress conditions: ergosterol (synthesized by yeast under aerobic conditions) and phytosterols (plant sterols imported by yeast cells from grape musts under anaerobiosis). Little is known about the physiological impact of phytosterols assimilation in comparison with ergosterol and the influence of sterol type on fermentation kinetics parameters. Moreover, studies to date have analyzed a limited number of yeast strains. Thus, the aim of this work was to compare the performances of a set of *Saccharomyces cerevisiae* wine strains that represent the diversity of industrial wine yeast, fermenting with phytosterols or ergosterol under two conditions: sterol limitation (sterol starvation) and high sugar content (the most common stress during fermentation). Results indicated that yeast cell viability was negatively impacted by both stressful conditions, resulting in sluggish and stuck fermentations. This study revealed the huge phenotype diversity of the *S. cerevisiae* strains tested, in particular in terms of cell viability. Indeed, strains with better viability maintenance completed fermentation earlier. Interestingly, we showed for the first time that sterol type differently affects a wide variety of phenotype, such as viability, biomass, fermentation kinetics parameters and biosynthesis of carbon central metabolism (CCM) metabolites. Ergosterol allowed preserving more viable cells at the end of fermentation and, as a consequence, a better completion of fermentation in both conditions tested, even if phytosterols also enabled the completion of alcoholic fermentation for almost all strains. These results highlighted the essential role of sterols during wine alcoholic fermentation to ensure yeast growth and avoid sluggish or stuck fermentations. Finally, this study emphasizes the importance of taking into account sterol types available during wine fermentation.

## Introduction

Sterols, along with phospholipids, sphingolipids and glycerolipids, are the major lipid components of the eukaryotic cell lipidome (Daum et al., 1998). In particular, sterols are responsible for regulating the fluidity, rigidity and permeability of cell membranes, being thus essential for eukaryotic cell viability (Rosenfeld et al., 2003; Henneberry and Sturley, 2005; Guan et al., 2009; Kodedová and Sychrová, 2015; Ermakova and Zuev, 2017). In yeast, sterols are mostly located in the plasma membrane. They are required for membrane structuring, initiation of cell growth, and regulation of ergosterol biosynthesis pathway genes expression (Smith et al., 1996; Leber et al., 2001; Klug and Daum, 2014). Ergosterol is the main yeast sterol (90% of the total content of sterols in Saccharomyces cerevisiae species; Rattray et al., 1975), followed by intermediates in the sterol biosynthetic pathway, such as zymosterol, fecosterol and episterol (Zinser et al., 1993). Phytosterols are plant sterols, the major ones being β-sitosterol, stigmasterol and campesterol (Nes, 1987). In grape berries, β-sitosterol represents between 85 and 90% of the total sterol content (Tumanov et al., 2015).

During alcoholic fermentation, Saccharomyces cerevisiae strains can synthesize, assimilate and accumulate significant amounts of sterols, which are associated with their growth, metabolism and viability (recently reviewed by Girardi Piva et al., 2022). They require oxygen to synthesize ergosterol and its precursors, as molecular O2 is the electron acceptor in the enzymatic steps of the sterol synthesis pathway catalyzed by Erg1p, Erg11p, Erg25p, Erg3p and Erg5p (Jordá and Puig, 2020). Under anaerobiosis, S. cerevisiae strains are capable of assimilating phytosterols from solid particles of grape must, thus restoring yeast cell growth (Casalta et al., 2012, 2013, 2019; Ochando et al., 2017).

Excessive grape must clarification during white wine production leads to the loss of solid particles with high lipid content, resulting in fermentations with low phytosterol content. This sterol limitation can lead to a high yeast cell mortality rate, a limited nitrogen assimilation and biomass production, and difficulties in completing alcoholic fermentation (Sablayrolles and Barre, 1986; Rodríguez-Vargas et al., 2007; Waldbauer et al., 2011; Casalta et al., 2016, 2019; Ochando et al., 2017).

Sterols are also essential for yeast adaptation to stress conditions during wine fermentation, such as ethanol stress (due to initial high sugar concentration; Chi and Arneborg, 1999; Aguilera et al., 2006). In the case of fermentation with high sugar content, which leads to high amounts of ethanol, the sterol content of the grape must is the key to maintain cell viability, and avoid incomplete fermentations. Indeed, ethanol concentrations higher than 10% v/v in the fermentation medium cause the diffusion of polar molecules from yeast cells, cellular ATP depletion and a decrease in membrane thickness and fluidity (Cartwright et al., 1986; Alexandre et al., 1994; Piper et al., 1997).

In the current literature, there is a lack of information about the impact of the sterol type on the ability of S. cerevisiae strains to perform wine fermentation. Indeed, previous studies either used ergosterol or phytosterols as a single sterol source or worked with both sterols in different concentrations, making difficult the comparison of the individual effect of each sterol (Luparia et al., 2004; Casalta et al., 2012, 2019; Duc et al., 2017; Ochando et al., 2017). In addition, these studies were carried out under specific fermentation conditions (different nitrogen, sterol and sugar levels) and for few strains.

Therefore, the aim of this work is to compare the impact of the sterol type (ergosterol versus phytosterols) on fermentation kinetics, cell viability, and Central Carbon Metabolism (CCM) metabolites for a wide set of 27 S. cerevisiae wine strains, representing the diversity of industrial wine yeast. Yeasts were evaluated under two typical stressful conditions encountered in oenology: sterol limitation, caused by a low sterol concentration in the grape must, as in the case of excessive clarification, and high sugar content, leading to a high production of ethanol during wine fermentation.

## Materials and methods

### Strains

We used a wide set of 27 *Saccharomyces cerevisiae* wine yeast strains, numbered L1 to L27, obtained as active dried yeasts (ADY) from Lallemand Œnologie (Blagnac, France), that represents the diversity of industrial wine yeast (Supplementary Figure 1). Fermenters were inoculated with 0.05 g/l of ADY, previously rehydrated for 20 min at 37°C in a glucose solution (50 g/l).

### Experimental fermentations

Experimental fermentations were performed with a synthetic must (SM), which mimics a grape must, following the protocol described by Bely et al. (1990). In this study, we used two different synthetic musts: SM 400 contained 400 mg/l of assimilable nitrogen, with a ratio (m/m) of 72% assimilable amino acids and 28% ammonium (NH_4_Cl) and 200 g/l of sugars (50% glucose and 50% fructose). SM 250 contained 250 mg/l of assimilable nitrogen, with the same ratio as SM400 of assimilable amino acids and ammonium (NH_4_Cl), and 260 g/l of sugars (50% glucose and 50% fructose). For sterol limitation condition, a high nitrogen content was used to provoke nitrogen-sterols imbalance, as described by Tesnière et al., 2013. The nitrogen content was reduced under the high sugar condition to be sure to assimilate all nitrogen content with the level of sterols used under this condition. So, in this last culture condition, the limiting nutrient is nitrogen and not sterols. The pH of both synthetic musts was adjusted to 3.3.

At first, sterol solutions with 15 g/l of sterols (phytosterols or ergosterol) containing Tween 80^®^ and ethanol (1:1, *v*/*v*) were prepared. Then, they were diluted with ethanol to obtain a final solution of 1.5 g/l sterols. A purified phytosterol complex, containing mainly β-sitosterol (≥ 70%; 85,451, Sigma-Aldrich) was used to prepare the phytosterol solution, while the ergosterol solution was prepared with synthetic ergosterol (E6510, Sigma-Aldrich).

To mimic sterol limitation, we used SM 400 and 1.0 mg/l of sterols (ergosterol or phytosterols). SM 250 and 5.0 mg/l of sterols (ergosterol or phytosterols) promoted high sugar condition. The experimental designs were built in order to compare both sterol types without comparing sterol limitation and high sugar conditions statistically (Table 1).

**Table 1:** Experimental designs: number of strains tested, synthetic must (SM) composition with assimilable nitrogen and total sugars and sterol type and concentration.

Strains	Condition	Synthetic must (SM) composition		Sterol		Assimilable nitrogen (mg/L)	Total sugars (g/L)	type	Concentration (mg/L)
27	Sterol limitation	400	200	Ergosterol	1.0
27	Sterol limitation	400	200	Phytosterols	1.0
27	High sugar content	250	260	Ergosterol	5.0
27	High sugar content	250	260	Phytosterols	5.0

All fermentations were performed in 300 ml fermenters filled with 250 ml of the corresponding medium. Fermenter medium deaeration was performed before sterol addition by bubbling pure argon for 20 min to ensure anaerobic conditions. Moreover, fermenters were fitted with fermentation locks to maintain anaerobiosis. All fermentations were performed in biological triplicates (total of 162 fermenters for each condition).

### Fermentation conditions

**Figure 1:**
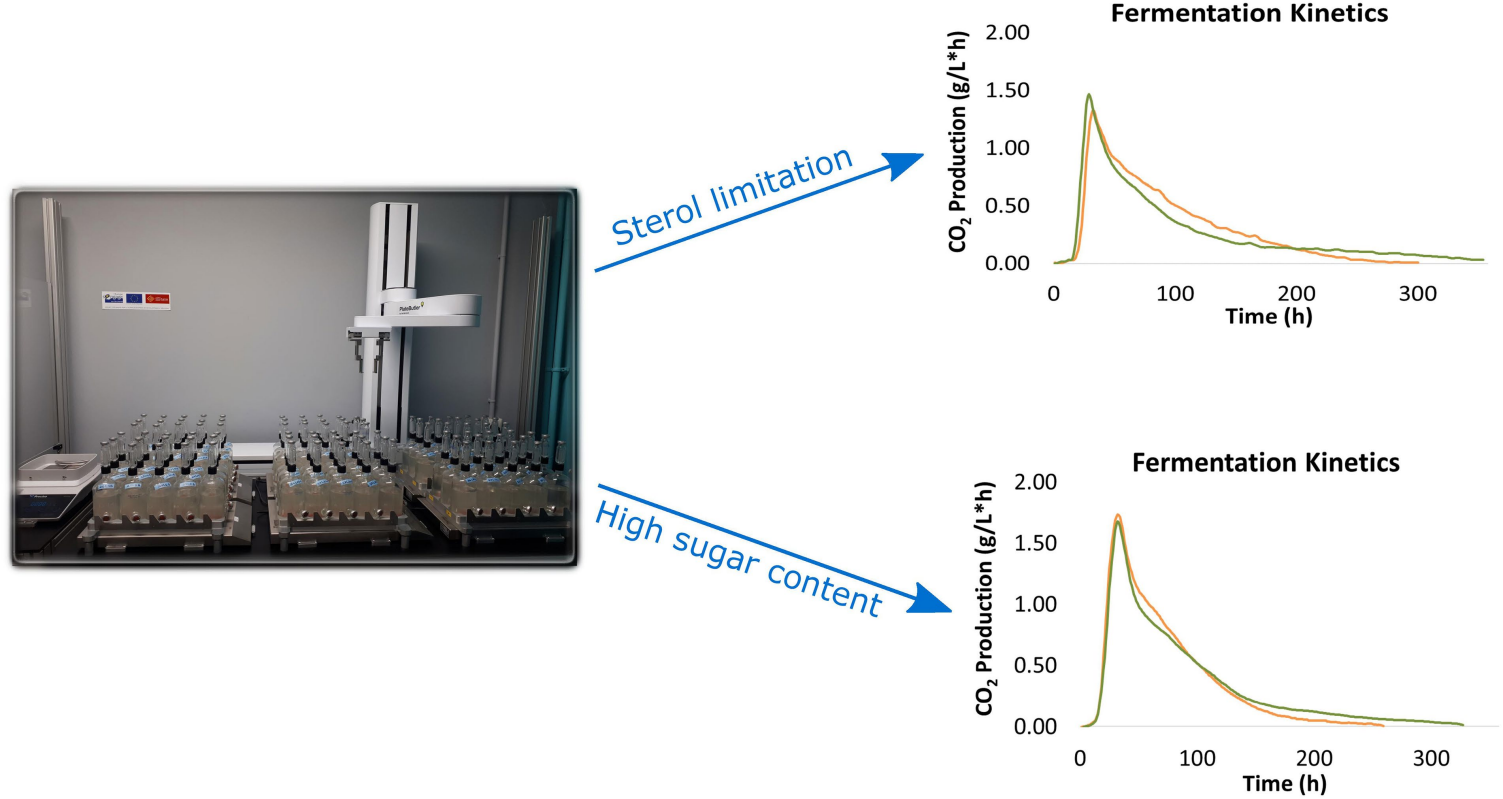
Automated robot system to follow fermentation progress (mechanical arm, stirring plates and precision balance) and example of fermentation kinetics obtained with an internally developed control software.

Fermenters were placed on magnetic stirring plates (260 rpm) at 24°C. In addition, fermentation kinetics were followed *via* an internally developed control software dedicated to the study of alcoholic fermentation with a temperature control system and automatic weighing. This task was performed with a robotic arm (LabServices, Breda, Netherlands) capable of moving the fermenters from their location on the stirring plates to a precision balance to measure their weight every hour (Figure 1). For each time point, an internally developed control software calculates 1/ the amount of produced CO_2_ (in g/L), which is proportional to the amount of sugars consumed, and 2/ the fermentation rate, which corresponds to the rate of CO_2_ production (in g CO_2_/Lh), proportional to the rate of sugar consumption.

### Sample preparation

Two samplings were done during both sets of experiments. The first sampling was done at 85% of the fermentation progress and the sample was divided in two: the first fraction was used for yeast cell viability and cell counting; the second one was centrifuged for 10 min at 3000 rpm at 4°C and the corresponding supernatant was stored at −20°C until nitrogen content analysis. The second sampling was collected at the end of fermentation. The centrifuged supernatant (10 min at 3000 rpm at 4°C) was stored at −20°C to quantify central carbon metabolism (CCM) metabolites.

### Analytical methods

#### Cell viability

Cell viability was determined by flow cytometry using an Accuri® C6 cytometer (Accuri, BD Biosciences). 1 ml of sample was centrifuged (3 min at 10,000 rpm, 4°C) and the cells were resuspended in the same volume of PBS (130 mM NaCl, 2.6 mM KCl, 7 mM Na_2_HPO_4_, 1.2 mM KH_2_PO_4_, pH 7.4). 450 μl of PBS and 5 μl of propidium iodide (0.1 mg/ml solution stored at 4°C protected from light; Calbiochem) were added in 50 μl of sample for the cell suspension. Samples were mixed by gentle shaking and propidium iodide (PI) flow cytometry analysis was performed 10 min after staining. Fluorescence data for cells stained by PI were collected in channel FL3 (670 nm LP with 488 nm laser). Viability was determined as the percentage of intact and fragile cells among all cells (Delobel et al., 2012).

#### Cell counting

Samples were diluted 1,600 fold with Isoton II® (Beckman Coulter). After sonication (30 s, 10 W), cells were counted with a Coulter Z2 electronic counter (Coulter Multisizer3, Beckman Coulter) fitted with a 100-μm aperture probe.

#### Nitrogen

The assimilated nitrogen content (ammonium and amino acids) was determined at 85% of fermentation progress. The ammonium (NH_4_) concentration was determined enzymatically (Boehringer Mannheim, Mannheim, Germany), as follows (Eq. 1):


$$\mathrm{Assimilated}\;NH4\;85\%=\left[NH4\right]\;\mathrm{must}-\left[NH4\right]\;85\%$$

The free amino acid (AA) content was determined by cation exchange chromatography with post-column ninhydrin derivatization (Biochrom 30, Biochrom), as described by Crépin et al. (2012). The assimilated amino acid content was determined as follows (Eq. 2):


$$\mathrm{Assimilated}\;\mathrm{AA}\;85\%=\left[\mathrm{AA}\right]\;\mathrm{must}-\left[\mathrm{AA}\right]\;85\%$$

#### Determination of CCM metabolites and residual sugars

Acetate, glycerol, succinate and residual sugars concentrations were determined by high-performance liquid chromatography (HPLC 1290 Infinity, Agilent Technologies, Santa Clara, CA, USA) with a Phenomenex Rezex ROA column (Agilent Technologies, Santa Clara, CA, USA) at 60°C, as described by Rollero et al. (2015). The column was eluted with 0.005 N H2SO4 at a flow rate of 0.6 ml/min. The acetic acid concentration was determined with a UV photometer at 210 nm and the concentrations of the other compounds were determined with a refractive index detector. Analysis was carried out with the Agilent EZChrom software package.

### Fermentation progress and variables coding

The fermentation progress corresponds to the ratio between the final CO_2_ production and the amount of produced CO_2_ at a specific time, which is proportional to the amount of sugars consumed. In this work, due to the different initial sugar concentrations in the two conditions, 85% of fermentation progress corresponded to 80 g/l of produced CO_2_ under sterol limitation and 100 g/l of produced CO_2_ under high sugar content. Similarly, 33% of fermentation progress corresponded to 30 g/l of produced CO_2_ under sterol limitation and 40 g/l of produced CO_2_ under high sugar content.

Some variables were coded to simplify results presentation: tCO2_x corresponded to the time to release “x” grams of CO_2_; tCO2_End corresponded to the time to achieve the end of fermentation; Vmax to the maximum fermentation rate.

### Statistical analysis

For each condition, statistical analyses were performed independently with R software version 3.6.2 (R Development Core Team, 2019). To describe the variability of the data set, PCA was performed with the package FactoMineR (v2.3). Strain and sterol factors evaluations were performed with two-way ANOVA (analysis of variance) using aov function with a statistical significance level of 0.5% after Bonferroni adjustment, following the model below (Eq. 3):


y=β0+β1Strain+β2Sterol+β12Strain∗Sterol+ϵ

Where ϵ are independent N(0, 𝜎^2^) error terms. Hypotheses were checked and the normality of residual distributions and homogeneity of variance were evaluated with standard diagnostic graphs.

The microsatellite genotypes of the 27 strains were obtained as described in Legras et al., 2007. The DC chord distance was calculated with a custom script. Clustering was performed using the neighbor module of the Philip package and trees drawn with Mega X fr Mac OS (Stecher et al., 2020).

## Results

A synthetic fermentation medium SM 400 with sterols at 1.0 mg/l allowed mimicking sterol deficiency conditions, due to a nitrogen/sterol imbalance (Tesnière et al., 2013). In parallel, fermentations with a balance between nitrogen and sterols content and with high sugar concentration (SM 250 with 5.0 mg/l of sterols) generated at first an osmotic stress and an ethanol stress throughout the fermentation progress. For both stressful conditions, we studied the impact of two sources of sterols: ergosterol and phytosterols under anaerobiosis for a set of 27 *Saccharomyces cerevisiae* strains. The monitoring of alcoholic fermentations enabled us to estimate the impact of sterol type on fermentation kinetics parameters maximum fermentation rate and times to achieve different g/L of released CO_2,_ *cf.* §2.6, and biological variables (yeast viability, biomass and nitrogen consumption). The influence of sterol compounds on CCM metabolites (acetate, glycerol, succinate and residual sugars) at the end of fermentation was evaluated simultaneously.

### Fermentations under sterol limitation

#### General description of biological, fermentation kinetic and CCM variables

Under sterol limitation, we showed that wine yeast strains did not have enough sterols to assimilate all nitrogen from the fermentation medium. Furthermore, we observed sluggish fermentations, despite the majority of strains being able to complete fermentation (except for L11, L19 and L21 that presented stuck fermentations, leaving more than 3.0 g/l residual sugars).

**Figure 2:**
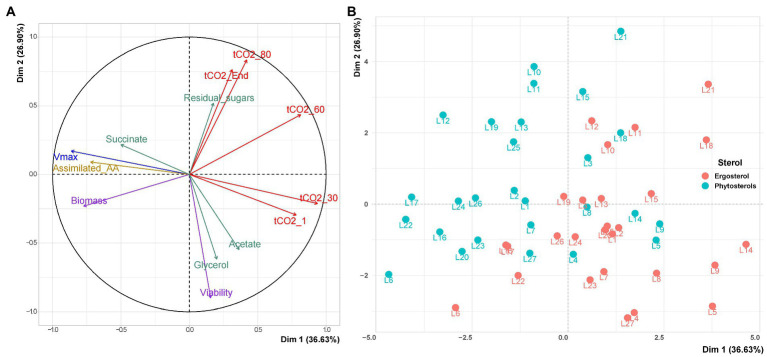
PCA for variables triplicate means of the 27 wine yeast strains under sterol limitation. **(A)** Projection of the variables used to describe fermentation kinetics, biological variables and central carbon metabolites on the 2 main components of PCA. The PCA variables are: maximum fermentation rate (Vmax), times to achieve 1, 30, 60, and 80 g/l of released CO_2_ and the end of fermentation (tCO2_1, tCO2_30, tCO2_60, tCO2_80 and tCO2_End, respectively); viability, biomass and assimilated amino acids at 85% of fermentation progress (Viability, Biomass and Assimilated_AA, respectively); acetate, glycerol, succinate and residual sugars at the end of fermentation. **(B)** Projection of the individuals.

A first Principal Component Analysis (PCA) was performed to detect the major variables, explaining the differences in the behavior of wine yeast strains utilizing ergosterol or phytosterols. Under sterol limitation, the two first principal components (Dim 1 and Dim 2) accounted for 63% of the total variation (Figure 2). Dim 1 was related to the start of fermentation (Vmax, biomass, tCO2_1 and tCO2_30) and Dim 2 to the fermentation achievement (Viability, tCO2_80 and tCO2_End).

Figure 2A shows that viability was negatively correlated with residual sugars and both tCO_2__End and tCO_2__80. Indeed, strains that displayed a higher viability at the end of alcoholic fermentation left less residual sugars and finished fermentation earlier. Moreover, viability was positively correlated with acetate and glycerol contents. Despite a direct correlation between Vmax, fermentation kinetics parameters at the beginning of fermentation (tCO_2__1 and tCO_2__30) and biomass production, there was no correlation between the maximum fermentation rate and fermentation kinetics parameters at the end of fermentation (tCO_2__End and tCO_2__80). In the same way, the quantity of living cells (Viability) was not related to this variable. This suggests that a faster start of fermentation is not related with a faster end of fermentation and a good maintenance of yeast cells viability.

As expected, the consumption of amino acids (assimilated_AA) and the maximum fermentation rate (Vmax) were correlated with the production of biomass.

Strains were able to assimilate all ammonium (data not shown). However, the consumption of amino acids was not complete (between 35 and 71%) and varied according to the *S. cerevisiae* strain and sterol type. Strains that consumed more amino acids were able to produce more biomass and achieved a higher maximum fermentation rate (Vmax) in little time (smaller tCO_2__1 and tCO_2__30). The graph of individuals performing with ergosterol or phytosterols showed a huge phenotype diversity of *S. cerevisiae* strains, since their dispersion according to the variables is quite high in Dim 1 and 2 (Figure 2B). Furthermore, it is possible to observe that the different strains tend to start fermentation faster with phytosterols and to better finish fermentation with ergosterol. However, a PCA is a descriptive analysis; it reveals the diversity of strains and highlights the most extreme behaviors (L6 that resisted better to sterol starvation than L21, for example, L6 having higher viability and shorter fermentation time than L21).

#### Impact of sterol type and strain diversity

The high diversity of response among the 27 *S. cerevisiae* strains found with the PCA analysis led us to evaluate the significance of the impact of strains and type of sterol with an analysis of variance. It was performed with the 10 most important variables under sterol limitation according to PCA results: fermentation kinetics variables (Vmax, time required for the production of 30 and 80 g/l of CO_2_ (tCO_2__30 and tCO_2__80, respectively)), biological variables at 85% of fermentation progress (viability, biomass and assimilated amino acids) and CCM variables at the end of fermentation. The *p* value threshold, the means and standard deviation (SD) for the 27 *S. cerevisiae* strains, sterol effect, strain effect and the interaction between both effects are shown in Table 2. Sterol effect evaluates whether there are significant differences between ergosterol and phytosterols; strain effect indicates whether the diversity among the 27 strains is statistically significant.

**Table 2:** Means, standard deviations (SD) and significance for the sterol limitation experiment.

Variable	Means ± SD (*n* = 27)		*p* value (*n* = 27)		Interaction
	Phytosterols	Ergosterol	Sterol effect	Strain effect
Vmax (g/L/h)	1.47 ± 0.15	1.36 ± 0.13	^***^	^***^	^**^
Viability (% living cells)	40.9 ± 15.8	49.0 ± 11.4	^***^	^***^	^***^
Biomass (cells/ mL)	7.41 × 10^7^ ± 1.67 × 10^7^	6.34 × 10^7^ ± 1.65 × 10^7^	^***^	^***^	^***^
Assimilated AA (mg/L)	126.6 ± 20.5	114.3 ± 16.8	^***^	^***^	^***^
tCO_2__30 (h)	41.0 ± 4.6	45.9 ± 4.8	^***^	^***^	NS
tCO_2__80 (h)	135.3 ± 29.1	126.0 ± 18.6	^***^	^***^	^***^
Acetate (g/L)	0.69 ± 0.10	0.85 ± 0.14	^***^	^***^	^***^
Glycerol (g/L)	5.80 ± 0.86	6.42 ± 1.05	^***^	^***^	^***^
Succinate (g/L)	0.65 ± 0.15	0.35 ± 0.16	^***^	^***^	^***^
Residual sugars (g/L)	1.32 ± 1.37	1.97 ± 2.58	^***^	^***^	^***^

Fermentation kinetic variables: maximum fermentation rate (Vmax), time to reach 30 (tCO2_30) and 80 g/l (tCO2_80) of released CO2; biological variables at 85% of fermentation progress: viability, yeast population and assimilated amino acids (assimilated AA); central carbon metabolism variables at the end of fermentation: acetate, glycerol and succinate and residual sugars. NS: not significant.

** *P* < 1.0 × 10^−3^;

*** *P* < 1.0 × 10^−4^

Our results show that the sterol type has a very significant effect on all fermentation kinetics, biological and CCM variables (value of *p* < 10^−4^). The strain effect was also very significant for these variables (value of *p*<10^−4^), confirming the phenotypic diversity of *S. cerevisiae* species in oenological fermentation under sterol starvation. However, not all strains displayed the same response to sterol type, as the interactions between sterol type and strain were highly significant for all variables (value of *p*<10^−3^), except tCO_2__30 (not significant).

**Figure 3:**
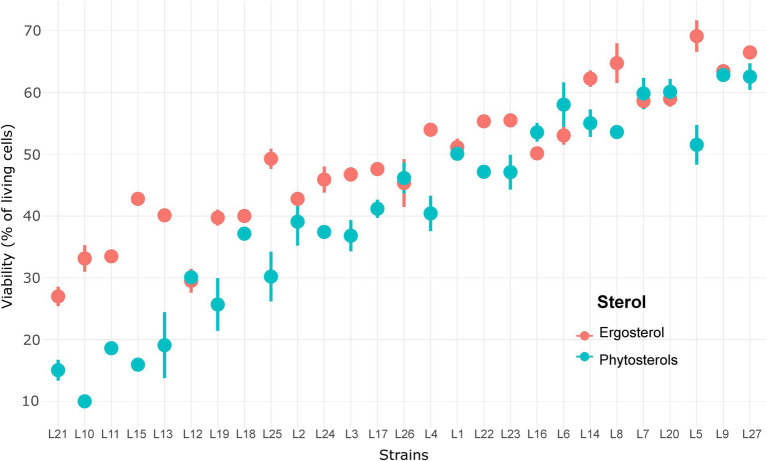
Viability of 27 *S. cerevisiae* strains under sterol limitation with ergosterol and phytosterols.

Interestingly, we observed an inversion of sterol type performance. In general, a faster start of fermentation and higher Vmax are observed with phytosterols, whereas ergosterol allows better yeast cell viability maintenance and, therefore, shorter times to produce 80 g/l of CO_2_. Strong interactions between sterol effect and strain effect indicate that some strains were more affected by the sterol type than others. Surprisingly, cell viability at 85% of fermentation progress (Figure 3) ranged between 10 and 70%, which clearly shows that sterol limitation induces high cell death. The lowest viabilities were noticed with phytosterols: less than 20% for strains L10, L11, L13, L15 and L21 (Figure 3). Indeed, the mean of living cells was higher with ergosterol (49.0 ± 11.4%) than with phytosterols (40.9 ± 15.8%). Moreover, it seems that the most resistant strains were also those least affected by the sterol type. These results confirm the diversity of *S. cerevisiae* response under sterol limitation and show strains’ sensitivity to sterol type.

In addition, a higher amino acid consumption led to more biomass production for strains performing with phytosterols. This led to start fermentation faster and a higher maximum fermentation rate: Vmax of 1.47 ± 0.15 g/l/h and tCO_2__30 of 41.0 ± 4.6 h with phytosterols, against a Vmax of 1.36 ± 0.13 g/l:h and a tCO_2__30 of 45.9 ± 4.8 h with ergosterol.

Regarding CCM metabolites, ergosterol resulted in an increase in acetate and glycerol and a decrease in succinate, compared with phytosterols (Table 2). Nevertheless, almost all strains were able to complete fermentation independently of sterol type, as residual sugars were less than 3.0 g/l with either sterols (except for L11, L19 and L21).

### Fermentations under high sugar content

#### General description of biological, fermentation kinetic and CCM variables

We then sought to evaluate the impact of the type of sterols in a medium presenting an excess of sugars on fermentation kinetic, biological and CCM variables. Under this condition, all strains consumed all the assimilable nitrogen (ammonium and amino acids). However, almost none of the wine strains could complete fermentation (only L5 was able to leave less than 3.0 g/l of residual sugars).

**Figure 4:**
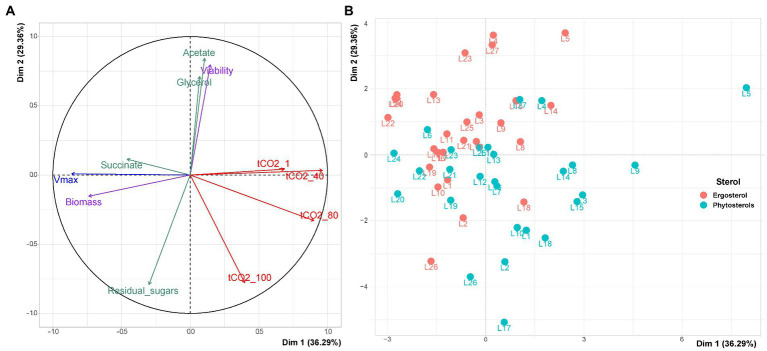
PCA for variables triplicate means of the 27 wine yeast strains under high sugars content. **(A)** Projection of the variables used to describe fermentation kinetics, biological variables and central carbon metabolites on the 2 main components of PCA. The PCA variables are: maximum fermentation rate (Vmax), times to achieve 1, 40, 80, and 100 g/l of released CO_2_ and the end of fermentation (tCO2_1, tCO2_40, tCO2_80, tCO2_100 and tCO2_End, respectively); viability and biomass at 85% of fermentation progress (Viability and Biomass, respectively); acetate, glycerol, succinate and residual sugars at the end of fermentation. **(B)** Projection of the individuals.

To have an overall view of our result, we first performed a PCA with these variables for the 27 *S. cerevisiae* strains for the two types of sterols: ergosterol and phytosterols (Figure 4). This PCA summarized 65% of the total variation in the two first dimensions.

As observed for the sterol limitation experiment, biomass production was positively correlated with the maximum fermentation rate and negatively correlated with times at the beginning of fermentation (tCO_2__1 and tCO_2__40). Indeed, strains that produced more cells started fermentation faster and had a better Vmax. Moreover, strains with high viability at 100 g/l of CO_2_ left less residual sugars and finished fermentation earlier (tCO_2__100).

Concerning MCC metabolites, acetate and glycerol were also strongly correlated with viability and not with succinate. Interestingly, similarly to sterol starvation, a fast start of the fermentation (Vmax and times to reach 1 or 40 g/l of CO_2_) was not associated with its rapid achievement (viability and the time to reach 100 g/l of CO_2_) under high sugar content.

In contrast to the situation of sterol starvation, when yeast strains were exposed to high sugars, the dispersion of the 27 strains (Figure 4B) shows that ergosterol lead to a better fermentation start than phytosterols (better Vmax and smaller tCO_2__1 and tCO_2__40). A better survival of yeast cells at the end of fermentation was also observed with ergosterol under this stress condition (as with strains L4, L5, L23 and L27, for example). Given the differences in strain responses s under these environments, it was then necessary to assess their significance.

#### Impact of sterol type and strain diversity

The effect of sterol type and strain were assessed with an analysis of variance (*cf* § 2.7) for 9 variables that described alcoholic fermentation: Vmax, viability, biomass, tCO2_40, tCO2_100 and CCM metabolites. These results are summarized in Table 3. We observed a high yeast strains effect for all variables tested (value of *p*<10^−4^). The sterol effect was also strong for all variables (value of *p*<10^−4^), except for the total cell population and residual sugars. Interestingly, ergosterol led to a better maximum fermentation rate and a reduced time to reach 40 g/l of CO_2_. Nonetheless, no significant difference was seen in the amount of biomass produced between ergosterol (10.41 × 10^7^ ± 2.17 × 10^7^ cells/mL) and phytosterols (10.34 × 10^7^ ± 2.23 × 10^7^ cells/mL).

**Table 3:** Means, standard deviations (SD) and significance for the high sugars content experiment.

Variable	Means ± SD (*n* = 27)		*p* value (*n* = 27)		Interaction
	Phytosterols	Ergosterol	Sterol effect	Strain effect
Vmax (g/L/h)	1.75 ± 0.20	1.82 ± 0.14	^***^	^***^	^***^
Viability (% living cells)	57.7 ± 18.8	63.1 ± 16.5	^***^	^***^	^***^
Biomass (cells/ mL)	10.34 × 10^7^ ± 2.23 × 10^7^	10.41 × 10^7^ ± 2.17 × 10^7^	NS	^***^	^***^
tCO_2__40 (h)	50.0 ± 7.5	46.4 ± 4.1	^***^	^***^	^***^
tCO_2__100 (h)	166.4 ± 36.4	139.4 ± 25.1	^***^	^***^	^***^
Acetate (g/L)	0.73 ± 0.12	0.87 ± 0.13	^***^	^***^	^***^
Glycerol (g/L)	6.69 ± 0.81	7.76 ± 0.91	^***^	^***^	^***^
Succinate (g/L)	2.37 ± 1.84	4.08 ± 2.03	^***^	^***^	^***^
Residual sugars (g/L)	22.4 ± 12.8	22.7 ± 13.0	NS	^***^	^***^

Fermentation kinetic variables: maximum fermentation rate (Vmax), time to reach 40 (tCO2_40) and 100 g/l (tCO2_100) of released CO2; biological variables at 85% of fermentation progress: viability and yeast population; central carbon metabolism variables at the end of fermentation: acetate, glycerol and succinate and residual sugars. *NS: not significant;*

*** *P* < 10^−4^

**Figure 5:**
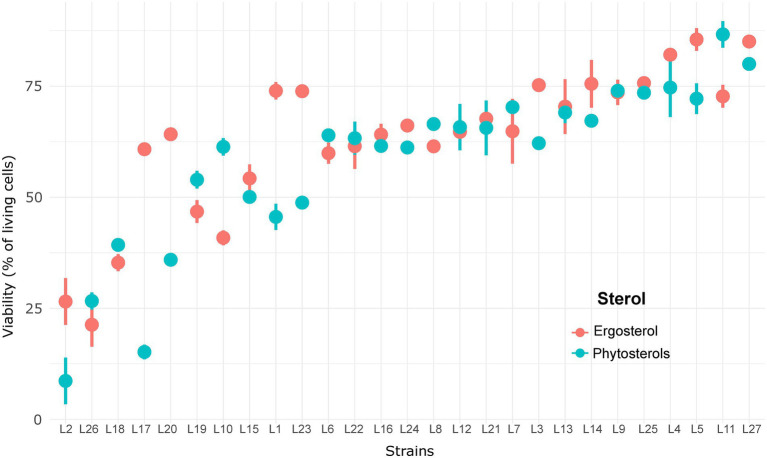
Viability of 27 *Saccharomyces cerevisiae* strains under high sugar content with ergosterol and phytosterols.

Ergosterol supply resulted in faster fermentations (139.4 ± 25.1 against 166.4 ± 36.4 h to reach 100 g/l of released CO_2_ with ergosterol and phytosterols, respectively) thanks to a better viability maintenance: 63.1 ± 16.5% of living cells with ergosterol and 57.7 ± 18.8% with phytosterols (Table 3). As shown in Figure 5, some strains better survived high ethanol levels: L3, L5, L14, L25 and L27 with ergosterol (75–85% of living cells) and L4, L5, L9, L11, L25 and L27 with phytosterols (73–87% of living cells). On the other hand, other strains faced difficulties to overcome this stress condition and had very low viabilities, between 21–41% with ergosterol (L2, L10, L18 and L26) and 8–36% with phytosterols (L2, L17, L20 and L26). Moreover, under this condition, strains were not able to consume all sugars from the synthetic medium (22.7 ± 13.0 g/l of residual sugars for fermentations with ergosterol and 22.4 ± 12.8 g/l with phytosterols). The presence of ergosterol resulted in increased synthesis of acetate and glycerol, compared to phytosterols (mean difference of 0.14 g/l for acetate and 1.07 g/l for glycerol).

## Discussion

In this work, we were interested in comparing the impact of two sterol types (phytosterols and ergosterol) in wine alcoholic fermentation for a large set of wine strains under two different stressful conditions, usually found in oenological fermentation contexts: limitation of sterols and high sugar content (which became an ethanol stress during fermentation).

### Sterol limitation

Phytosterols appeared to be more effective at the beginning of fermentation, whereas ergosterol increased long-term viability and therefore, led to shorter fermentation time. Based on these results, we can hypothesize that ergosterol, as the native yeast sterol, could better maintain the integrity of yeast membranes than phytosterols, as it preserves more viable cells until the end of fermentation, resulting in a faster fermentation. These results confirm the hypothesis formulated by Luparia et al. (2004), who assumed ergosterol to be more effective in maintaining viability during fermentation than phytosterols, although such hypothesis has been put forward without comparing both sterols at the same concentration. Thus, we can hypothesize that yeast membranes would be better maintained with an ergosterol supply, as the replacement of ergosterol by phytosterols would change the membrane lateral pressure profile (distribution of lateral stresses across the width of the lipid bilayer), which is associated with its elasticity. Indeed, Samuli Ollila et al. (2007) showed that variations in the type of sterol (replacement of cholesterol by its precursor) resulted in modifications in the lateral pressure profile of different membrane systems.

In this research work, we observed a high variability for cell viability under sterol limitation, with cell viability rate being as low as 15% for the most sensitive strains (Figure 3). These results suggest that a lack of sterols provokes damages in membrane functionality, such as a decrease in membrane thickness and rigidity, resulting in yeast cell death (Abe and Hiraki, 2009; Duc et al., 2017; Ermakova and Zuev, 2017). Interestingly, this sensitivity to sterol limitation is highly dependent on the strain, and of the sterol type. It also seems that the less resistant strains are also the most affected by the type of sterols. This suggests that yeast strains have different abilities to cope with the substitution of ergosterol by phytosterols.

Differences between the lipid content in *S. cerevisiae* yeast cells membrane and their sterol requirement could explain the phenotype diversity under sterol limitation highlighted in this study. Another hypothesis could be the diversity of expression of genes associated with phytosterols uptake and sterol assimilation among our set of *S. cerevisiae* strains during fermentation, such as the ABC transporters *AUS1* and *PDR11* (Duc et al., 2020; Tesnière et al., 2021). Indeed, Tesnière et al. (2021) found discrepancies in sterol uptake between 13 *S. cerevisiae* strains (from different ecological niches), showing the impact of yeasts’ genetic background in the assimilation of sterols.

If we turn to CCM metabolites production, it can be noted that acetate production varied as a function of the wine strain and increased in the presence of ergosterol, in comparison with phytosterols. This observation suggests that acetate synthesis could be linked to initial yeast lipid storage, yeast strain assimilation efficiency or to the available sterol type.

**Figure 6:**
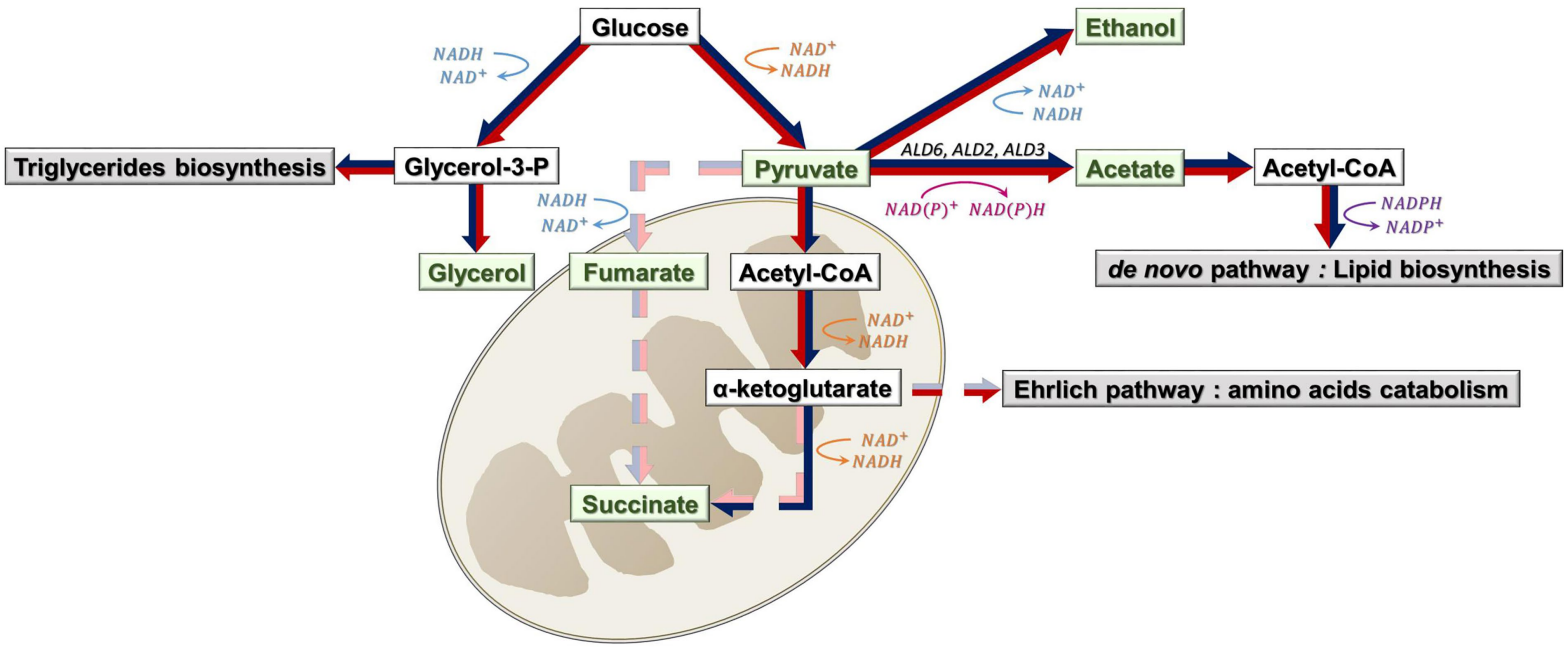
Biosynthesis of CCM metabolites (green) and associated pathways (gray) under sterol limitation and high sugar content. Possibly active pathways under sterol limitation are represented with red arrows; Possibly active pathways under high sugar content are represented with dark blue arrows; possibly inactive pathways are represented with dashed arrows (red for sterol limitation and blue for high sugar content). Reduction reactions are in orange and pink; oxidation reactions are in blue and purple. *ALD6*, *ALD2* and *ALD3* are genes involved in acetate synthesis.

Sterol starvation also impacted the final glycerol content, which was correlated with acetate (Figure 2). This could be explained by an activation of triglycerides biosynthesis, to protect yeast membranes. Triglycerides biosynthesis requires the synthesis of its intermediate, glycerol-3-phosphate (Figure 6). Thus, the excess of glycerol-3-phosphate produced would be converted to glycerol (Ochando et al., 2017). Moreover, as we have shown previously, glycerol is positively correlated with viability. We can then hypothesize that, for our set of strains, a higher glycerol synthesis could better protect cells from sterol limitation and allow a better support of yeast cell viability, in particular with ergosterol as sterol source.

During fermentation, succinate can be synthesized through two different branches: the reductive pathway (where fumarate is reduced to succinate), which is responsible for around 75% of its synthesis, and the oxidative pathway (Camarasa et al., 2003). In our case, we can suppose that pyruvate is drawn toward lipid synthesis and, as a consequence, less pyruvate is available for succinate synthesis through the reductive pathway.

Regarding the oxidative pathway, we could hypothesize that a higher flux of α-ketoglutarate to the Ehrlich pathway would occur for catabolizing amino acids in excess (from nitrogen-lipid imbalance), and thus less available for succinate synthesis (Ochando et al., 2017). This could explain the low succinate contents observed in our experiments (whereas this acid is usually found between 0.2 and 1.5 g/l in wines and between 0.2 and 0.7 at the end of fermentations with synthetic musts; Whiting, 1976; Ochando et al., 2017; Deroite et al., 2018).

We also showed that succinate was correlated with biomass production, as both parameters are dependent on the amount of nitrogen consumed, whose assimilation is dependent on the quantity of available sterols (Tesnière et al., 2013; Duc et al., 2017; Ochando et al., 2017). Indeed, we confirmed that sterol starvation affected nitrogen assimilation, as a significant amino acid fraction was not consumed during wine fermentation (Table 2). Moreover, a significant strain effect for biomass production was observed, which could be explained by different nitrogen and sterol requirements among the 27 *S. cerevisiae* strains (Brice et al., 2014).

Another important finding of this work was the fact that, under sterol limitation, phytosterols can partly replace ergosterol and enable most strains to achieve complete fermentation, in contrast to Luparia et al. (2004) findings. The explanation for this divergence lies in the different concentration between ergosterol and phytosterols used by Luparia et al. (2004).

### Fermentations under high sugar content

The most remarkable result from fermentations under high sugar content experiment was the very clear differences induced by the replacement of ergosterol by phytosterols for most fermentation kinetics, biological variables and CCM metabolites. Interestingly, performances of yeasts with ergosterol were better than phytosterols for all kinetics and biological parameters under this condition. In particular, ergosterol allowed better viability maintenance and consequently shorter fermentation time. Indeed, ergosterol supplementation limits interdigitation and maintains yeast membrane thickness and fluidity in the presence of ethanol (Landolfo et al., 2010; Vanegas et al., 2012). Thus, we could hypothesize that *S. cerevisiae* strains tested with a higher cell lipid content, in particular ergosterol content, should be more resistant to ethanol stress (Novotný et al., 1992; Aguilera et al., 2006; Turanlı-Yıldız et al., 2017). Moreover, ergosterol, being the native sterol in yeasts, probably helps in maintaining a better membrane integrity compared to phytosterols whose spatial conformations are different (Samuli Ollila et al., 2007).

MCC metabolites were also affected by the excess of sugars in the synthetic must and the consequent increase in ethanol synthesis. The glycerol synthesis observed under this condition could be explained by its essential function in restoring the normal biological activities of yeast cells under ethanol stress and its osmoregulation role (Hohmann, 1997; Nevoigt and Stahl, 1997). Thus, this could explain its positive correlation with viability (Figure 4): the most ethanol-tolerant strains were able to synthesize more glycerol and to maintain higher viability during fermentation. Consequently, shorter fermentation times were observed for these strains.

Acetate synthesis was also correlated with glycerol (Figure 4). Glycerol synthesis implies an NAD+ release, while acetate synthesis enzymes can consume NAD+ (Ald2p and Ald3p) or NADP+ (Ald5p and Ald6p). Studies have shown that acetic acid formation has been linked to the up-regulation of *ALD2* and *ALD3* genes and down-regulation of *ALD6*, in response to the redox imbalance caused by glycerol formation (Navarro-Aviño et al., 1999; Bro et al., 2003). Thus, the hypothesis to explain pyruvate flow to acetate synthesis mostly by Ald2p and Ald3p and a down-regulation of *ALD6* under ethanol stress would be a compensation of the redox imbalance. Moreover, amino acid catabolism by the Ehrlich pathway would be less active under this condition, as all amino acids were consumed. As a consequence, succinate would be synthesized through the reductive pathway, thanks to ɑ-ketoglutarate availability (Figure 6).

## Conclusion

The originality of the current study was to investigate how *S. cerevisiae* wine strains are impacted during alcoholic fermentation by two sterol sources: natural yeast sterol (ergosterol) and grape phytosterols. This has been analyzed from wine fermentation parameters, yeast cell viability and CCM metabolites, relying on a large set of wine strains under two different conditions: high sugars content and sterol limitation. Sterol starvation limited nitrogen assimilation, and thus limited yeast multiplication and resulted in a viability decrease and sluggish fermentation. High sugar concentration led to an increase in ethanol content, characterized by a loss of viability at the end of fermentation and by incomplete fermentations.

A remarkable result was the high loss of viable cells under both conditions. However, yeasts responded differently to each condition tested. Under sterol limitation, this high cell death resulted from yeast inability to trigger an appropriate stress response. Under high sugar content, sterols could contribute to a better yeast cell adaptation to high levels of ethanol (as shown by the fact that viability was generally higher at 85% of fermentation progress under high sugar content compared to sterol limitation), but this was not sufficient to enable fermentation completion. In addition, we observed significant differences between ergosterol and phytosterols on yeast growth and cell survival, as well as fermentation kinetics parameters and CCM metabolites under both stress conditions. The most relevant finding of this study was the better capacity of ergosterol, over phytosterols, to maintain a better viability at the end of fermentation under both conditions tested. A higher maximum fermentation rate and a faster start of fermentation were observed with phytosterols under sterol limitation and with ergosterol under high sugar content. Moreover, an increase in acetate and glycerol synthesis was seen with ergosterol under both conditions compared to the phytosterols condition. The impact of sterols in acetate synthesis highlighted the importance of sterols for avoiding undesirable organoleptic quality in wines due to an excessive amount of acetate.

Another striking finding was the huge phenotype diversity of the 27 *S. cerevisiae* under both stress conditions, suggesting that sterols uptake mechanisms and associated genetic regulations varied in function of *S. cerevisiae* strains tested and their yeast cell lipid composition.

This work highlights the essential role of sterols during wine fermentation for nitrogen assimilation, biomass production, maintaining good membrane integrity and functionality and, consequently, avoiding sluggish and stuck fermentations. From a practical point of view, our results indicate that, depending on the fermentation conditions, some strains will be more adapted than others to fermentation, based on their sterol use and their capacity to survive under stress conditions. Ultimately, this might become an important criterion in wine yeast choice, particularly considering climate change and thus more challenging conditions.

In the future, it will be interesting to investigate gene expression and quantify strains cell lipid content to better understand sterols biosynthesis, uptake and assimilation, which could explain both *S. cerevisiae* phenotype diversity under conditions tested in this study and the better yeast resistance to these conditions with ergosterol as sterol type.

## Data availability statement

The original contributions presented in the study are included in the article/Supplementary material, further inquiries can be directed to the corresponding author.

## Author contributions

J-RM, VG, CT, J-LL, DF, EC, and AO-J, contributed to the conception and design of the study. TN performed the statistical analysis strategy. FM and MP performed HPLC and amino acids analysis, respectively. GG implemented all experiments, samplings, sample preparation, and ammonium analysis and drafted the manuscript. All authors contributed to the article and approved the submitted version.

## Funding

The authors declare that this study received funding from Lallemand Œnologie.

## Conflict of interest

The authors declare that the research was conducted in the absence of any commercial or financial relationships that could be construed as a potential conflict of interest.

## Publisher’s note

All claims expressed in this article are solely those of the authors and do not necessarily represent those of their affiliated organizations, or those of the publisher, the editors and the reviewers. Any product that may be evaluated in this article, or claim that may be made by its manufacturer, is not guaranteed or endorsed by the publisher.
