# Supplementary material for: Influence of ergosterol and phytosterols on wine alcoholic fermentation with Saccharomyces cerevisiae strains
Source: Front Microbiol. 2022 Sep 8;13:966245. doi: 10.3389/fmicb.2022.966245 (PMC9493300; doi:10.3389/fmicb.2022.966245)
Supplement: Supplementary file 1 [file Data_Sheet_1.docx]

Supplementary Material

# Supplementary Figures and Tables

## Supplementary Figures


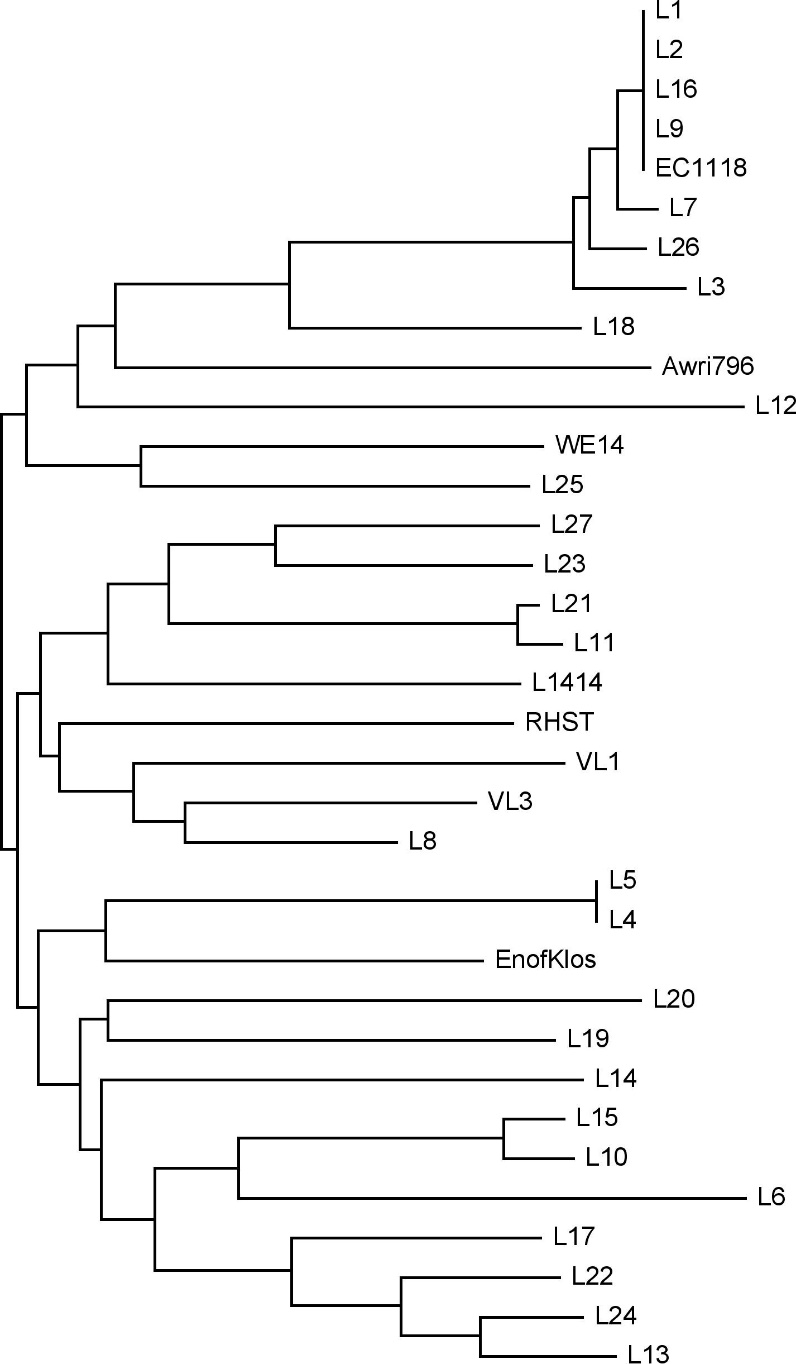


**Supplementary Figure 1.** Neighbor joining tree presenting the genetic relatedness between 27 strains compared to 8 industrial wine starters (EC1118, AWRI796, WE14, L1414, Enoferm Klosterneubourg, RHST, VL1, VL3) estimated from to their micro satellite profile. Pairwise distance has been estimated according to the DC chord distance.

**B**

**A**


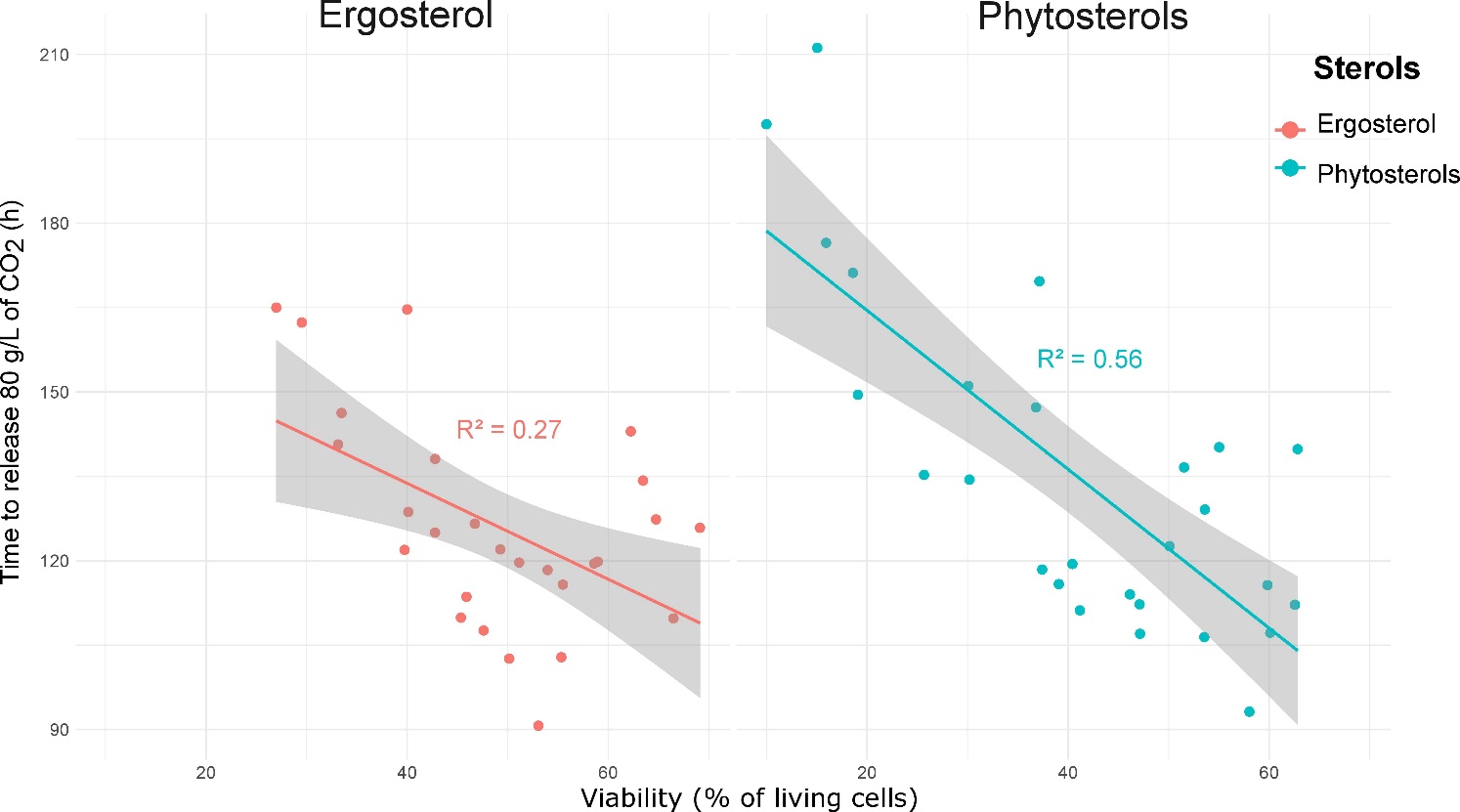


**Supplementary Figure 2.** Relation between viability and time to reach 80 g/L of released CO_2_ for sterol limitation experiment for 27 *S. cerevisiae* strains with ergosterol **(A)** and phytosterols **(B)**.

**
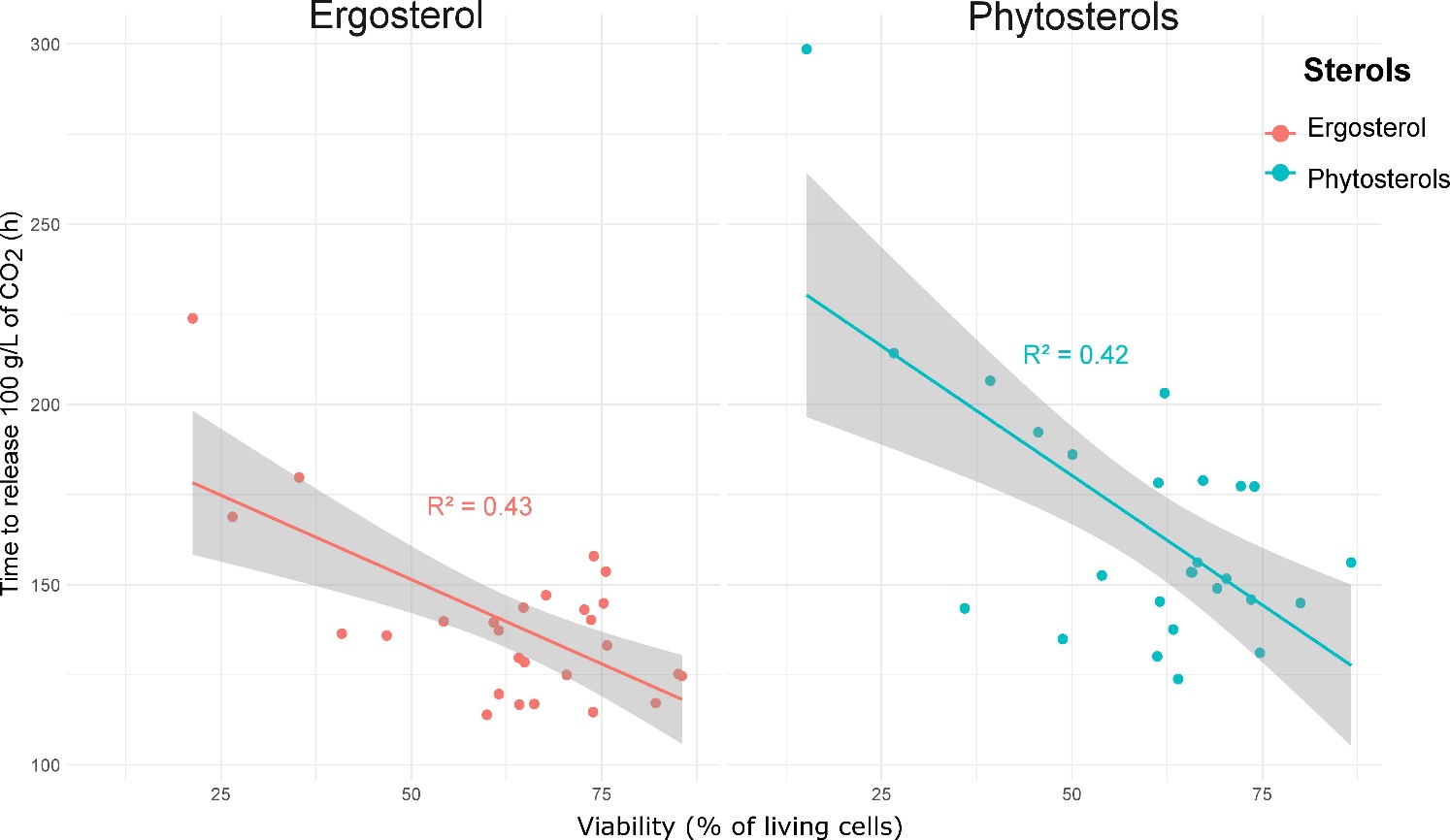
**

**A**

**B**

**Supplementary Figure 3.** Relation between viability and time to reach 100 g/L of released CO_2_ for high sugars content experiment for 27 *S. cerevisiae* strains with ergosterol **(A)** and phytosterols **(B)**.

## Supplementary Tables

**Supplementary Table 1.** Means and standard deviations for sterol limitation experiments for 27 *S. cerevisiae* strains with ergosterol. Fermentation kinetic variables: maximum fermentation rate (Vmax), time to reach 30 (tCO2_30) and 80 g/L (tCO2_80) of released CO2; biological variables at 85% of fermentation progress: viability, yeast biomass and assimilated amino acids (assimilated AA); central carbon metabolism variables at the end of fermentation: acetate, glycerol and succinate and residual sugars (Res sugars).

| **Strain** | **Vmax**  **(g/L*h)** | **Viability**  **(% living cells)** | **Biomass (cells/mL)** | **AA cons (mg/L)** | **tCO_2__30**  **(h)** | **tCO_2__80**  **(h)** | **Acetate**  **(g/L)** | **Glycerol**  **(g/L)** | **Succinate (g/L)** | | **Res sugars**  **(g/L)** |
| --- | --- | --- | --- | --- | --- | --- | --- | --- | --- | --- | --- |
| L1 | 1.27 ± 0.02 | 51.1 ± 1.4 | 6.02E+07 ± 0.00E+00 | 105.4 ± 4.9 | 46.1 ± 0.8 | 119.7 ± 1.9 | 0.7 ± 0.0 | 5.5 ± 0.1 | | 0.2 ± 0.0 | 1.1 ± 0.2 |
| L2 | 1.35 ± 0.04 | 42.8 ± 0.8 | 6.52E+07 ± 0.00E+00 | 138.8 ± 8.0 | 48.7 ± 2.5 | 125.0 ± 5.6 | 0.8 ± 0.0 | 6.9 ± 0.1 | | 0.3 ± 0.0 | 1.3 ± 0.6 |
| L3 | 1.36 ± 0.02 | 46.7 ± 1.2 | 7.87E+07 ± 1.16E+07 | 118.3 ± 3.7 | 44.1 ± 0.6 | 126.6 ± 2.6 | 0.8 ± 0.0 | 5.9 ± 0.1 | | 0.4 ± 0.0 | 0.6 ± 0.1 |
| L4 | 1.41 ± 0.04 | 54.0 ± 0.7 | 7.35E+07 ± 2.05E+07 | 114.0 ± 13.8 | 48.1 ± 1.1 | 118.3 ± 3.7 | 1.2 ± 0.0 | 9.2 ± 0.1 | | 0.3 ± 0.0 | 0.4 ± 0.0 |
| L5 | 1.2 ± 0.03 | 69.1 ± 2.5 | 3.96E+07 ± 0.00E+00 | 100.9 ± 15.2 | 54.0 ± 1.1 | 125.8 ± 2.4 | 1.0 ± 0.0 | 9.0 ± 0.2 | | 0.4 ± 0.0 | 0.4 ± 0.0 |
| L6 | 1.57 ± 0.17 | 53.1 ± 1.5 | 9.83E+07 ± 1.64E+07 | 157.6 ± 12.6 | 39.8 ± 0.9 | 94.3 ± 6.2 | 0.9 ± 0.1 | 7.6 ± 0.5 | | 0.3 ± 0.0 | 0.3 ± 0.1 |
| L7 | 1.23 ± 0.03 | 58.6 ±0.6 | 9.84E+07 ± 0.00E+00 | 99.5 ± 6.4 | 47.7 ± 0.9 | 119.5 ± 2.7 | 0.8 ± 0.0 | 5.8 ± 0.1 | | 0.3 ± 0.0 | 0.6 ± 0.0 |
| L8 | 1.14 ± 0.07 | 64.7 ±3.2 | 9.04E+07 ± 0.00E+00 | 97.2 ± 4.3 | 51.1 ± 2.1 | 124.5 ± 5.0 | 0.8 ± 0.0 | 6.0 ± 0.0 | | 0.2 ± 0.0 | 0.6 ± 0.1 |
| L9 | 1.08 ± 0.05 | 63.5 ± 0.9 | 5.49E+07 ± 0.39E+07 | 106.2 ± 18.0 | 54.5 ± 1.6 | 134.3 ± 2.5 | 1.0 ± 0.1 | 6.5 ± 0.5 | | 0.3 ± 0.0 | 0.6 ± 0.1 |
| L10 | 1.42 ± 0.01 | 33.1 ± 2.1 | 5.77E+07 ±  0.23E+07 | 124.7 ± 0.8 | 44.8 ± 0.5 | 140.7 ± 2.8 | 0.7 ± 0.0 | 5.6 ± 0.1 | | 0.3 ± 0.3 | 2.7 ± 0.9 |
| L11 | 1.41 ± 0.01 | 33.5 ± 0.3 | 5.70E+07 ± 0.00E+00 | 107.6 ± 3.4 | 44.9 ± 0.8 | 146.3 ± 1.6 | 0.8 ± 0.0 | 5.5 ± 0.0 | | 0.4 ± 0.1 | 6.9 ± 0.8 |
| L12 | 1.56 ± 0.06 | 29.5 ± 1.9 | 5.52E+07 ± 0.96E+07 | 127.1 ± 12.8 | 41.3 ± 1.3 | 162.3 ±10.3 | 0.8 ± 0.0 | 5.8 ± 0.1 | | 0.1 ± 0.2 | 2.9 ± 0.3 |
| L13 | 1.43 ± 0.04 | 40.1 ± 0.6 | 5.89E+07 ± 0.00E+00 | 115.8 ± 2.9 | 45.0 ± 0.9 | 128.7 ± 5.9 | 0.8 ± 0.0 | 6.7 ± 0.0 | | 0.4 ± 0.2 | 2.0 ± 1.6 |
| L14 | 1.16 ± 0.02 | 62.2 ± 1.3 | 4.01E+07 ± 0.00E+00 | 101.3 ± 4.3 | 56.7 ± 0.5 | 143.0 ± 1.4 | 0.9 ± 0.0 | 7.0 ± 0.0 | | 0.7 ± 0.1 | 0.8 ± 0.1 |
| L15 | 1.27 ± 0.03 | 42.8 ± 0.9 | 5.36E+07 ± 0.00E+00 | 113.0 ± 2.2 | 48.5 ± 0.9 | 138.1 ± 2.7 | 0.8 ± 0.0 | 5.8 ± 0.1 | | 0.4 ± 0.0 | 0.8 ± 0.1 |
| L16 | 1.47 ± 0.04 | 50.2 ± 0.8 | 7.67E+07 ± 0.00E+00 | 126.6 ± 7.2 | 41.1 ± 0.3 | 102.6 ± 1.3 | 0.8 ± 0.0 | 5.2 ± 0.0 | | 0.7 ± 0.1 | 1.0 ± 0.0 |
| L17 | 1.44 ± 0.07 | 47.6 ± 0.5 | 6.85E+07 ± 0.00E+00 | 153.1 ± 3.9 | 41.8 ± 0.8 | 107.4 ± 1.4 | 0.6 ± 0.0 | 6.9 ± 0.1 | | 0.5 ± 0.0 | 2.3 ± 0.6 |
| L18 | 1.19 ± 0.03 | 40.0 ± 1.1 | 3.39E+07 ± 0.00E+00 | 128.0 ± 1.3 | 53.8 ± 1.2 | 166.3 ± 4.1 | 0.6 ± 0.0 | 5.8 ± 0.2 | | 0.5 ± 0.0 | 0.5 ± 0.1 |
| L19 | 1.47 ± 0.03 | 39.7 ± 1.3 | 7.56E+07 ± 0.00E+00 | 113.6 ± 3.7 | 41.0 ± 1.0 | 120.6 ± 3.3 | 0.9 ± 0.0 | 6.0 ± 0.1 | | 0.3 ± 0.0 | 3.9 ± 3.8 |
| L20 | 1.34 ± 0.03 | 59.0 ± 1.3 | 5.95E+07 ± 0.00E+00 | 108.4 ± 6.3 | 43.1 ± 1.3 | 119.8 ± 3.0 | 1.0 ± 0.0 | 6.8 ± 0.1 | | 0.5 ± 0.0 | 5.2 ± 0.6 |
| L21 | 1.31 ± 0.05 | 27.0 ±1.5 | 3.82E+07 ± 0.00E+00 | 90.6 ± 2.7 | 46.6 ± 1.0 | 164.6 ± 3.4 | 0.9 ± 0.0 | 5.5 ± 0.1 | | 0.2 ± 0.0 | 12.2 ± 1.1 |
| L22 | 1.44 ± 0.02 | 55.3 ± 0.6 | 7.28E+07 ± 0.00E+00 | 126.6 ± 6.3 | 39.2 ± 0.6 | 102.8 ± 0.8 | 0.9 ± 0.0 | 6.2 ± 0.0 | | 0.2 ± 0.2 | 1.0 ± 0.1 |
| L23 | 1.41 ± 0.05 | 55.5 ± 0.9 | 6.52E+07 ± 0.00E+00 | 101.2 ± 10.4 | 44.1 ± 1.5 | 115.8 ± 7.8 | 1.0 ± 0.0 | 7.9 ± 0.2 | | 0.7 ± 0.1 | 0.8 ± 0.3 |
| L24 | 1.39 ± 0.03 | 45.9 ± 2.1 | 5.89E+07 ± 0.00E+00 | 115.5 ± 7.0 | 41.4 ± 0.8 | 113.6 ± 2.3 | 0.9 ± 0.0 | 6.8 ± 0.1 | | 0.3 ± 0.3 | 0.9 ± 0.1 |
| L25 | 1.38 ± 0.01 | 49.3 ± 1.6 | 5.89E+07 ± 0.00E+00 | 97.5 ± 5.5 | 45.7 ± 0.8 | 122.0 ± 2.2 | 0.7 ± 0.0 | 5.1 ± 0.1 | | 0.2 ± 0.0 | 0.7 ± 0.1 |
| L26 | 1.42 ± 0.03 | 43.0 ± 3.9 | 6.74E+07 ± 0.09E+07 | 98.1 ± 1.0 | 41.8 ± 0.7 | 109.9 ± 1.6 | 0.8 ± 0.0 | 5.5 ± 0.1 | | 0.1 ± 0.1 | 2.0 ± 0.3 |
| L27 | 1.48 ± 0.02 | 66.5 ± 1.1 | 5.57E+07 ± 0.25E+07 | 99.9 ± 0.7 | 45.8 ± 0.4 | 109.8 ± 2.0 | 1.1 ± 0.0 | 6.8 ± 0.3 | | 0.1 ± 0.2 | 0.7 ± 0.3 |

**Supplementary Table 2.** Means and standard deviations for sterol limitation experiments for 27 *S. cerevisiae* strains with phytosterols. Fermentation kinetic variables: maximum fermentation rate (Vmax), time to reach 30 (tCO_2__30) and 80 g/L (tCO_2__80) of released CO2; biological variables at 85% of fermentation progress: viability, yeast biomass and assimilated amino acids (assimilated AA); central carbon metabolism variables at the end of fermentation: acetate, glycerol and succinate and residual sugars (Res sugars).

| **Strain** | **Vmax**  **(g/L*h)** | **Viability**  **(% living cells)** | **Biomass (cells/mL)** | **AA cons (mg/L)** | **tCO_2__30**  **(h)** | **tCO_2__80**  **(h)** | **Acetate (g/L)** | **Glycerol**  **(g/L)** | **Succinate (g/L)** | **Res sugars**  **(g/L)** |
| --- | --- | --- | --- | --- | --- | --- | --- | --- | --- | --- |
| L1 | 1.42 ± 0.07 | 50.1 ± 0.6 | 7.65E+07 ± 0.54 E+07 | 112.1 ± 5.4 | 40.7 ± 2.0 | 122.6 ± 7.7 | 0.6 ± 0.0 | 5.1 ±0.1 | 0.5 ± 0.0 | 0.8 ± 0.1 |
| L2 | 1.49 ± 0.02 | 39.1 ± 3.9 | 6.97E+07 ± 0.46 E+07 | 148.0 ± 10.1 | 42.2 ± 3.3 | 115.8 ± 1.9 | 0.7 ± 0.0 | 6.2 ± 0.1 | 0.5 ± 0.0 | 3.0 ± 1.1 |
| L3 | 1.29 ± 0.07 | 36.8 ± 2.5 | 6.40E+07 ± 0.00E+00 | 99.0 ± 7.1 | 42.5 ± 1.6 | 147.3 ± 6.3 | 0.7 ± 0.0 | 5.3 ± 0.0 | 0.4 ± 0.1 | 0.9 ± 0.5 |
| L4 | 1.44 ± 0.07 | 40.4 ± 2.9 | 5.96E+07 ± 0.00E+00 | 121.3 ± 0.6 | 42.9 ± 2.0 | 114.8 ± 8.1 | 0.9 ± 0.0 | 7.9 ± 0.1 | 0.6 ± 0.0 | 0.2 ± 0.0 |
| L5 | 1.17 ± 0.03 | 51.5 ± 3.2 | 4.29E+07 ± 0.03E+07 | 98.3 ± 1.4 | 50.8 ± 1.8 | 140.4 ± 6.6 | 0.8 ± 0.0 | 7.8 ± 0.1 | 0.6 ± 0.0 | 0.1 ± 0.0 |
| L6 | 1.63 ± 0.10 | 58.0 ± 3.6 | 1.09E+08 ± 0.01E+08 | 153.0 ± 3.3 | 36.8 ± 1.6 | 95.9 ± 5.4 | 0.8 ± 0.0 | 7.4 ± 0.2 | 1.1 ± 0.1 | 0.1 ± 0.0 |
| L7 | 1.40 ± 0.09 | 59.8 ± 2.5 | 7.59E+07 ± 0.00E+00 | 113.8 ± 13.2 | 41.3 ± 2.0 | 115.7 ± 6.4 | 0.7 ± 0.0 | 5.1 ± 0.1 | 0.5 ± 0.0 | 0.3 ± 0.0 |
| L8 | 1.26 ± 0.07 | 53.6 ± 0.7 | 7.01E+07 ± 0.51E+07 | 102.2 ± 3.9 | 45.8 ± 1.8 | 129.1 ± 5.0 | 0.6 ± 0.0 | 5.2 ± 0.0 | 0.5 ± 0.0 | 0.3 ± 0.0 |
| L9 | 1.13 ± 0.04 | 62.8 ± 0.4 | 6.43E+07 ± 0.00E+00 | 86.0 ± 1.3 | 50.2 ± 1.0 | 139.8 ± 1.8 | 0.7 ± 0.0 | 5.4 ± 0.1 | 0.5 ± 0.0 | 0.2 ± 0.0 |
| L10 | 1.56 ± 0.05 | 10.0 ± 1.1 | 6.64E+07 ± 0.26E+07 | 139.3 ± 0.8 | 39.2 ± 1.4 | 197.6 ± 11.0 | 0.6 ± 0.0 | 5.3 ± 0.2 | 0.6 ± 0.0 | 0.3 ± 0.1 |
| L11 | 1.56 ± 0.04 | 18.6 ± 0.8 | 6.49E+07 ± 0.26E+07 | 128.2 ± 5.1 | 39.7 ± 0.8 | 171.2 ± 11.4 | 0.7 ± 0.0 | 5.2 ± 0.0 | 0.8 ± 0.0 | 3.2 ± 0.4 |
| L12 | 1.79 ± 0.06 | 30.1 ± 0.8 | 6.72E+07 ± 0.00E+00 | 147.6 ± 7.4 | 33.9 ± 0.8 | 151.1 ± 6.6 | 0.6 ± 0.0 | 5.2 ± 0.1 | 0.6 ± 0.0 | 1.8 ± 0.3 |
| L13 | 1.53 ± 0.06 | 19.1 ± 5.3 | 8.01E+07 ± 0.80E+07 | 120.9 ± 5.8 | 40.1 ± 1.4 | 149.5 ± 11.1 | 0.7 ± 0.0 | 6.0 ± 0.1 | 0.7 ± 0.0 | 3.7 ± 1.4 |
| L14 | 1.25 ± 0.03 | 55.0 ± 2.2 | 6.19E+07 ± 0.19E+07 | 116.8 ± 3.4 | 48.9 ± 0.6 | 140.2 ± 2.9 | 0.7 ± 0.0 | 6.2 ± 0.0 | 0.6 ± 0.0 | 1.9 ± 2.0 |
| L15 | 1.40 ± 0.06 | 15.9 ± 0.6 | 6.31E+07 ± 0.00E+00 | 123.2 ± 1.1 | 43.3 ± 1.3 | 176.5 ± 10.7 | 0.7 ± 0.0 | 5.3 ± 0.0 | 0.6 ± 0.0 | 2.3 ± 0.3 |
| L16 | 1.57 ± 0.05 | 53.6 ± 1.5 | 1.14E+08 ± 0.17E+08 | 137.7 ± 6.1 | 37.5 ± 0.7 | 106.4 ± 1.8 | 0.7 ± 0.0 | 5.0 ± 0.0 | 0.5 ± 0.0 | 0.4 ± 0.0 |
| L17 | 1.57 ± 0.06 | 41.2 ± 1.5 | 8.45E+07 ± 0.90E+07 | 175.8 ± 6.0 | 36.4 ± 1.0 | 111.2 ± 4.9 | 0.5 ± 0.0 | 6.2 ± 0.1 | 0.8 ± 0.0 | 0.5 ± 0.0 |
| L18 | 1.29 ± 0.04 | 37.2 ± 1.0 | 5.41E+07 ± 1.09E+07 | 145.7 ± 6.3 | 48.4 ± 0.6 | 169.7 ± 2.5 | 0.5 ± 0.0 | 5.8 ± 0.0 | 0.5 ± 0.0 | 0.4 ± 0.3 |
| L19 | 1.56 ± 0.03 | 25.7 ± 4.3 | 7.80E+07 ± 0.54E+07 | 123.0 ± 8.0 | 37.0 ± 0.7 | 135.3 ± 6.0 | 0.7 ± 0.0 | 5.4 ± 0.0 | 0.7 ± 0.0 | 5.4 ± 2.6 |
| L20 | 1.59 ± 0.05 | 60.1 ± 2.1 | 8.03E+07 ± 0.65E+07 | 126.4 ± 7.9 | 35.8 ± 0.7 | 107.2 ± 3.3 | 0.9 ± 0.0 | 6.1 ± 0.1 | 0.8 ± 0.0 | 1.1 ± 0.0 |
| L21 | 1.42 ± 0.05 | 15.1 ± 1.7 | 5.89E+07 ± 0.05E+07 | 106.7 ± 6.7 | 42.6 ± 1.2 | 211.2 ± 8.4 | 0.7 ± 0.0 | 5.2 ± 0.0 | 0.8 ± 0.0 | 3.4 ± 1.5 |
| L22 | 1.62 ± 0.01 | 47.2 ± 0.6 | 1.01E+08 ± 0.00E+00 | 154.8 ± 9.5 | 35.1 ± 0.1 | 107.0 ± 1.1 | 0.7 ± 0.0 | 5.6 ± 0.1 | 0.8 ± 0.0 | 0.6 ± 0.0 |
| L23 | 1.56 ± 0.06 | 47.1 ± 2.8 | 8.17E+07 ± 0.66E+07 | 134.5 ± 2.3 | 39.3 ± 1.2 | 112.3 ± 6.7 | 0.8 ± 0.0 | 6.8 ± 0.1 | 0.8 ± 0.0 | 0.4 ± 0.0 |
| L24 | 1.54 ± 0.05 | 37.4 ± 0.7 | 8.10E+07 ± 0.00E+00 | 147.2 ± 6.3 | 37.1 ± 1.0 | 120.1 ± 4.3 | 0.8 ± 0.0 | 6.4 ± 0.1 | 0.7 ± 0.0 | 1.0 ± 0.1 |
| L25 | 1.54 ± 0.06 | 30.2 ± 4.0 | 7.66E+07 ± 0.04E+07 | 114.9 ± 14.2 | 39.6 ± 1.0 | 134.4 ± 6.4 | 0.6 ± 0.0 | 4.7 ± 0.0 | 0.5 ± 0.0 | 1.8 ± 0.6 |
| L26 | 1.5 ± 0.05 | 46.2 ± 2.5 | 9.83E+07 ± 0.00E+00 | 118.5 ± 1.2 | 38.1 ± 0.6 | 114.0 ± 2.2 | 0.7 ± 0.0 | 5.1 ± 0.0 | 0.5 ± 0.0 | 1.3 ± 0.2 |
| L27 | 1.54 ± 0.1 | 62.6 ± 2.1 | 5.76E+07 ± 0.14E+07 | 123.1 ± 25.7 | 42.8 ± 2.3 | 116.4 ± 8.6 | 0.8 ± 0.0 | 6.0 ± 0.2 | 0.8 ± 0.0 | 0.1 ± 0.0 |

**Supplementary Table 3.** Means and standard deviations for high sugar content experiments for 27 *S. cerevisiae* strains with ergosterol. Fermentation kinetic variables: maximum fermentation rate (Vmax), time to reach 40 (tCO_2__40) and 100 g/L (tCO_2__100) of released CO_2_; biological variables at 85% of fermentation progress: viability and yeast biomass; central carbon metabolism variables at the end of fermentation: acetate, glycerol and succinate and residual sugars (Res sugars).

| **Strain** | **Vmax**  **(g/L*h)** | **Viability**  **(% living cells)** | **Biomass**  **(cells/mL)** | **tCO_2__40**  **(h)** | **tCO_2__100**  **(h)** | **Acetate**  **(g/L)** | **Glycerol**  **(g/L)** | **Succinate**  **(g/L)** | **Res sugars**  **(g/L)** |
| --- | --- | --- | --- | --- | --- | --- | --- | --- | --- |
| L1 | 1.74 ± 0.04 | 74.0 ± 2.0 | 1.13E+08 ± 0.07E+08 | 45.2 ± 0.4 | 157.9 ± 6.9 | 0.7 ± 0.0 | 6.9 ± 0.1 | 4.5 ± 0.1 | 41.2 ± 1.4 |
| L2 | 1.99 ± 0.01 | 26.5 ± 5.3 | 8.06E+07 ± 0.04E+07 | 46.9 ± 1.0 | 168.8 ± 12.3 | 0.6 ± 0.0 | 7.8 ± 0.0 | 5.8 ± 0.4 | 42.1 ± 4.9 |
| L3 | 1.76 ± 0.02 | 75.2 ± 1.0 | 1.05E+08 ± 0.03E+08 | 44.9 ± 0.5 | 144.8 ± 3.3 | 0.9 ± 0.0 | 7.3 ± 0.0 | 2.1 ± 0.0 | 7.8 ± 1.7 |
| L4 | 1.74 ± 0.05 | 82.1 ± 0.9 | 8.88E+07 ± 0.02E+07 | 49.2 ± 1.2 | 117.2 ± 2 .2 | 1.0 ± 0.0 | 9.6 ± 0.1 | 4.0 ± 0.3 | 6.2± 1.3 |
| L5 | 1.47 ± 0.03 | 85.5 ± 2.5 | 6.56E+07 ±0.50E+07 | 55.4 ± 1.2 | 124.7 ± 4.2 | 1.0 ± 0.0 | 9.9 ± 0.2 | 2.5 ± 0.2 | 0.7 ± 0.3 |
| L6 | 1.95 ± 0.06 | 59.9 ± 2.4 | 1.38E+08 ± 0.07E+08 | 41.9 ± 0.9 | 113.9 ± 4.0 | 0.8 ± 0.0 | 9.3 ± 0.1 | 4.3 ± 0.1 | 14.0 ± 2.4 |
| L7 | 1.81 ± 0.04 | 64.9 ± 7.3 | 1.04E+08 ± 0.08E+08 | 45.2 ± 1.2 | 128.5 ± 2.0 | 0.8 ± 0.0 | 6.9 ± 0.2 | 4.7 ± 0.2 | 31.4 ± 1.2 |
| L8 | 1.61 ± 0.04 | 61.5 ± 0.7 | 6.71E+07 ± 0.01E+07 | 50.1 ± 1.5 | 137.3 ± 5.7 | 0.8 ± 0.0 | 7.5 ± 0.1 | 2.5 ± 0.1 | 24.9 ± 1.1 |
| L9 | 1.71 ± 0.04 | 73.6 ± 2.8 | 1.12E+08 ± 0.00E+00 | 51.6 ± 1.0 | 140.3 ± 4.6 | 0.9 ± 0.0 | 7.5 ± 0.0 | 3.3 ± 0.1 | 20.4 ± 4.3 |
| L10 | 1.87 ± 0.04 | 40.9 ± 1.7 | 1.07E+08 ± 0.01E+08 | 44.3 ± 0.8 | 136.4 ± 4.7 | 0.8 ± 0.0 | 6.9 ± 0.0 | 2.2 ± 0.0 | 36.6 ± 1.0 |
| L11 | 1.99 ± 0.03 | 72.7 ± 2.6 | 9.64E+07 ± 0.02E+07 | 42.8 ± 0.8 | 143.1 ± 2.6 | 0.9 ± 0.0 | 6.9 ± 0.1 | 3.1 ± 0.0 | 24.1 ± 1.4 |
| L12 | 1.88 ± 0.05 | 64.7 ± 3.6 | 9.97E+07 ± 0.02E+07 | 41.0 ± 0.9 | 143.7 ± 7.9 | 0.8 ± 0.0 | 7.3 ± 0.1 | 2.9 ± 0.1 | 31.9 ± 2.4 |
| L13 | 1.96 ± 0.03 | 70.4 ± 6.2 | 1.09E+08 ± 0.05E+08 | 44.1 ± 0.6 | 125.0 ± 5.2 | 0.9 ± 0.0 | 8.2 ± 0.0 | 6.0 ± 0.1 | 14.6 ± 2.2 |
| L14 | 1.71 ± 0.03 | 75.5 ± 5.4 | 7.51E+07 ± 0.14E+07 | 54.6 ± 1.2 | 153.7 ± 4.3 | 1.1 ± 0.1 | 8.2 ± 0.1 | 5.7 ± 0.2 | 23.8 ± 2.8 |
| L15 | 1.59 ± 0.04 | 54.3 ± 3.2 | 1.07E+08 ± 0.00E+00 | 49.9 ± 0.9 | 139.8 ± 0.3 | 1.1 ± 0.0 | 7.7 ± 0.0 | 1.9 ± 0.2 | 7.4 ± 0.9 |
| L16 | 1.79 ± 0.04 | 64.1 ± 2.4 | 1.31E+08 ± 0.04E+08 | 45.9 ± 1.0 | 129.8 ± 5.6 | 0.8 ± 0.0 | 6.9 ± 0.1 | 4.1 ± 0.2 | 30.5 ± 1.5 |
| L17 | 1.7 ± 0.01 | 60.8 ± 0.3 | 1.22E+08 ± 0.07E+08 | 47.8 ± 0.3 | 139.0 ± 1.0 | 0.7 ± 0.0 | 8.1 ± 0.0 | 2.9 ± 0.0 | 12.8 ± 0.0 |
| L18 | 1.72 ± 0.04 | 35.3 ± 1.9 | 9.08E+07 ± 0.17E+07 | 53.8 ± 1.0 | 179.8 ± 9.8 | 0.8 ± 0.0 | 7.6 ± 0.2 | 6.5 ± 0.2 | 38.2 ± 0.2 |
| L19 | 1.88 ± 0.01 | 46.8 ± 2.6 | 1.11E+08 ± 0.03E+08 | 42.8 ± 0.3 | 135.9 ± 3.3 | 0.8 ± 0.0 | 7.1 ± 0.1 | 3.5 ± 0.2 | 32.9 ± 2.4 |
| L20 | 1.96 ± 0.03 | 64.2 ± 1.5 | 1.02E+08 ± 0.01E+08 | 42.8 ± 0.5 | 116.8 ± 1.1 | 1.0 ± 0.0 | 8.1 ± 0.0 | 11.3 ± 0.2 | 30.8 ± 0.7 |
| L21 | 1.99 ± 0.04 | 67.7 ± 2.7 | 8.98E+07 ± 0.01E+07 | 44.4 ± 0.8 | 147.1 ± 1.4 | 0.9 ± 0.0 | 6.8 ± 0.1 | 3.0 ± 0.1 | 26.5 ± 2.0 |
| L22 | 1.88 ± 0.02 | 61.5 ± 5.1 | 1.56E+08 ± 0.01E+08 | 41.3 ± 0.7 | 119.7 ± 6.3 | 0.9 ± 0.0 | 7.6 ± 0.1 | 4.3 ± 0.2 | 19.5 ± 5.3 |
| L23 | 1.92 ± 0.01 | 73.9 ± 1.2 | 8.27E+07 ± 0.04E+07 | 45.5 ± 0.0 | 114.7 ± 2.0 | 1.0 ± 0.0 | 9.2 ± 0.0 | 3.0 ± 0.1 | 13.9 ± 3.1 |
| L24 | 1.95 ± 0.01 | 66.1 ± 1.5 | 1.38E+08 ± 0.00E+00 | 41.8 ±0.3 | 116.9 ± 2.9 | 0.9 ± 0.0 | 8.1 ± 0.1 | 6.1 ± 0.3 | 15.5 ± 3.4 |
| L25 | 1.90 ±0.05 | 75.7 ± 1.2 | 1.06E+08 ± 0.00E+00 | 45.6 ± 0.8 | 129.4 ± 6.9 | 0.8 ± 0.0 | 6.7 ± 0.0 | 0.9 ± 0.0 | 8.3 ± 0.2 |
| L26 | 1.82 ± 0.05 | 21.3 ± 4.9 | 1.24E+08 ± 0.01E+08 | 44.5 ± 0.9 | 234.9 ± 20.1 | 0.7 ± 0.0 | 6.7 ± 0.1 | 5.1 ± 0.0 | 50.4 ± 1.1 |
| L27 | 1.91 ± 0.04 | 85.1 ± 1.2 | 8.98E+07 ± 0.25E+07 | 48.4 ±1.4 | 125.3 ± 3.1 | 1.1 ± 0.0 | 8.4 ± 0.0 | 3.8 ± 0.2 | 6.4 ± 0.6 |

**Supplementary Table 4.** Means and standard deviations for high sugar content experiments for 27 *S. cerevisiae* strains with phytosterols. Fermentation kinetic variables: maximum fermentation rate (Vmax), time to reach 40 (tCO_2__40) and 100 g/L (tCO_2__100) of released CO_2_; biological variables at 85% of fermentation progress: viability and yeast biomass; central carbon metabolism variables at the end of fermentation: acetate, glycerol and succinate and residual sugars (Res sugars).

| **Strain** | **Vmax**  **(g/L*h)** | **Viability**  **(% living cells)** | **Biomass**  **(cells/mL)** | **tCO_2__40**  **(h)** | **tCO_2__100**  **(h)** | **Acetate**  **(g/L)** | **Glycerol**  **(g/L)** | **Succinate**  **(g/L)** | **Res sugars**  **(g/L)** |
| --- | --- | --- | --- | --- | --- | --- | --- | --- | --- |
| L1 | 1.65 ± 0.03 | 45.6 ± 2.9 | 9.79E+07 ± 0.03E+07 | 50.8 ± 0.5 | 192.3 ± 4.3 | 0.6 ± 0.0 | 6.3 ± 0.01 | 1.7 ± 0.0 | 30.9 ± 2.9 |
| L2 | 1.9 ± 0.04 | 8.7± 5.2 | 8.86E+07 ± 0.15E+07 | 52.6 ± 0.7 | NA | 0.4 ± 0.0 | 7.2 ± 0.01 | 3.5 ± 0.2 | 48.4 ± 0.9 |
| L3 | 1.59 ± 0.04 | 62.2 ± 1.1 | 8.81E+07± 0.06E+07 | 52.9 ± 0.8 | 203.2 ± 3.1 | 0.8 ± 0.0 | 6.3 ± 0.2 | 1.3 ± 0.1 | 11.4 ± 2.4 |
| L4 | 1.71 ± 0.06 | 70.7 ± 6.6 | 1.02E+08 ± 0.23E+08 | 54.9 ± 1.6 | 131.1 ± 8.0 | 0.9 ± 0.0 | 7.8 ± 0.2 | 1.9 ± 0.3 | 7.8 ± 1.4 |
| L5 | 1.05 ± 0.03 | 72.2 ± 3.5 | 4.10E+07 ± 0.09E+07 | 77.3 ± 0.7 | 183.3 ± 1.2 | 1.1 ± 0.1 | 9.4 ± 0.1 | 0.8 ± 0.0 | 0.3 ± 0.0 |
| L6 | 1.91 ± 0.08 | 63.9 ± 1.1 | 1.52E+08 ± 0.01E+08 | 45.0 ± 0.9 | 123.8 ± 4.4 | 0.6 ± 0.0 | 8.3 ± 0.2 | 3.2 ± 0.1 | 8.1 ± 0.7 |
| L7 | 1.73 ± 0.03 | 70.3 ± 1.1 | 1.20E+08 ± 0.16E+08 | 50.7 ± 1.4 | 151.7 ± 4.7 | 0.7 ± 0.0 | 6.1 ± 0.1 | 2.6 ± 0.1 | 26.2 ± 1.5 |
| L8 | 1.52 ± 0.03 | 66.5± 0.8 | 8.92E+07 ± 0.14E+07 | 56.8 ± 1.0 | 156.2 ± 9.0 | 0.7 ± 0.0 | 6.6 ± 0.2 | 1.3 ± 0.5 | 13.3 ± 2.8 |
| L9 | 1.43 ± 0.02 | 74.0 ± 0.8 | 7.40E+07 ± 0.10E+07 | 61.4 ± 1.5 | 177.3 ± 1.2 | 0.8 ± 0.1 | 6.5 ± 0.4 | 0.9 ± 0.1 | 12.8 ± 1.8 |
| L10 | 1.84 ± 0.03 | 61.4 ± 2.0 | 1.05E+08 ± 0.00E+00 | 50.9 ± 0.8 | 178.3 ± 4.4 | 0.6 ± 0.0 | 5.7 ± 0.1 | 1.2 ± 0.0 | 36.1 ± 2.3 |
| L11 | 1.93 ± 0.04 | 86.7 ± 3.0 | 1.08E+08 ± 0.16E+08 | 46.7 ± 0.3 | 156.2 ± 4.7 | 0.8 ± 0.1 | 6.1 ± 0.3 | 2.1 ± 0.3 | 17.1 ± 5.0 |
| L12 | 1.84 ± 0.04 | 65.8 ± 5.2 | 1.05E+08 ± 0.09E+08 | 45.7 ± 1.1 | 153.4 ± 9.0 | 0.7 ± 0.1 | 6.3 ± 0.3 | 2.0 ± 0.5 | 21.8 ± 9.3 |
| L13 | 1.9 ± 0.03 | 69.1 ± 2.4 | 1.16E+08 ± 0.07E+08 | 49.5 ± 0.8 | 149.0 ± 3.8 | 0.8 ± 0.0 | 6.9 ± 0.1 | 2.5 ± 0.0 | 18.9 ± 2.9 |
| L14 | 1.72 ± 0.05 | 67.2 ± 0.8 | 8.69E+07 ± 0.23E+07 | 55.8 ± 2.3 | 178.9 ± 3.4 | 0.8 ± 0.0 | 7.0 ± 0.2 | 4.8 ± 0.4 | 26.2 ± 1.5 |
| L15 | 1.54 ± 0.03 | 50.1 ± 1.0 | 8.59E+07 ± 2.83E+07 | 53.7 ± 0.2 | 175.3 ± 12.5 | 0.8 ± 0.0 | 6.2 ± 0.1 | 1.1 ± 0.2 | 24.6 ± 3.3 |
| L16 | 1.71 ± 0.04 | 61.6 ± 1.2 | 1.17E+08 ± 0.02E+08 | 48.2 ± 3.6 | 155.6 ± 17.5 | 0.7 ± 0.0 | 6.0 ± 0.1 | 1.6 ± 0.1 | 21.3 ± 2.4 |
| L17 | 1.69 ± 0.03 | 15.2± 1.6 | 1.23E+08 ± 0.03E+08 | 48.3 ± 1.3 | 298.6 ± 20.2 | 0.6 ± 0.0 | 6.6 ± 0.1 | 1.9 ± 0.0 | 44.8 ± 3.1 |
| L18 | 1.64 ± 0.05 | 39.3 ± 0.7 | 8.95E+07 ± 0.02E+07 | 52.5 ± 1.6 | 206.6 ± 6.2 | 0.6 ± 0.0 | 6.9 ± 0.1 | 3.1 ± 0.1 | 32.5 ± 1.0 |
| L19 | 1.87 ± 0.06 | 54.0 ± 2.0 | 1.01E+08 ± 0.11E+08 | 42.8 ± 1.2 | 152.6 ± 25.6 | 0.7 ± 0.0 | 6.1 ± 0.0 | 1.6 ± 0.0 | 32.1 ± 1.8 |
| L20 | 1.94 ± 0.03 | 35.9 ± 0.5 | 1.07E+08 ± 0.00E+08 | 42.4 ± 0.3 | 143.4 ± 0.1 | 0.7 ± 0.0 | 6.7 ± 0.2 | 10.3 ± 0.4 | 37.6 ± 0.6 |
| L21 | 1.99 ± 0.01 | 65.6 ± 6.2 | 1.03E+08 ± 0.16E+08 | 42.9 ± 1.2 | 153.5 ± 26.5 | 0.8 ± 0.1 | 6.0 ± 0.5 | 2.0 ± 0.5 | 21.7 ± 5.7 |
| L22 | 1.84 ± 0.03 | 63.3 ± 3.7 | 1.37E+08 ± 0.02E+08 | 42.0 ± 0.4 | 137.6 ± 5.6 | 0.7 ± 0.0 | 6.3 ± 0.1 | 1.9 ± 0.0 | 22.1 ± 4.7 |
| L23 | 1.92 ± 0.01 | 48.8 ± 0.9 | 9.91E+07 ± 0.54E+07 | 44.8 ± 0.3 | 134.9 ± 2.1 | 0.8 ± 0.0 | 7.3 ± 0.1 | 2.4 ± 0.1 | 18.6 ± 2.3 |
| L24 | 1.95 ± 0.01 | 61.2 ± 0.4 | 1.47E+08 ± 0.06E+08 | 40.6 ± 0.2 | 130.1 ± 4.8 | 0.7 ± 0.0 | 6.9 ± 0.0 | 3.2 ± 0.3 | 18.6 ± 5.9 |
| L25 | 1.82 ± 0.04 | 73.5 ± 0.6 | 9.94E+07 ± 0.87E+07 | 44.5 ± 1.8 | 145.9 ± 17.9 | 0.8 ± 0.1 | 6.0 ± 0.03 | 0.8 ± 0.1 | 7.5 ± 7.1 |
| L26 | 1.74 ± 0.06 | 26.6 ± 1.9 | 1.19E+08 ± 0.00E+08 | 46.3 ± 1.3 | 214.3 ± 7.0 | 0.6 ± 0.0 | 6.0 ± 0.01 | 2.0 ± 0.2 | 42.7 ± 0.8 |
| L27 | 1.84 ± 0.04 | 80.0 ± 1.2 | 9.19E+07 ± 0.04E+08 | 49.9 ± 0.8 | 144.9 ± 1.1 | 0.9 ± 0.0 | 7.1 ± 0.0 | 2.4 ± 0.1 | 1.0 ± 0.3 |
